# A large language model-based approach to quantifying the effects of social determinants in liver transplant decisions

**DOI:** 10.1038/s41746-025-02025-y

**Published:** 2025-11-17

**Authors:** Emily Robitschek, Asal Bastani, Kathryn Horwath, Savyon Sordean, Mark J. Pletcher, Jennifer C. Lai, Sergio Galletta, Elliott Ash, Jin Ge, Irene Y. Chen

**Affiliations:** 1 University of California, San Francisco, California USA; 2 ETH Zurich, Zurich, Switzerland; 3 University of California, Berkeley, California USA

**Keywords:** Health policy, Health services

## Abstract

Psychosocial risk factors and social determinants of health (SDOH) contribute to persistent disparities in liver transplantation access. We developed a large language model framework to extract and analyze how these factors influence care trajectories. Prevalence of key modifiable barriers varied by demographics: social support gaps (35.4%, disproportionately affecting females), recent substance use (14.2–22.7%), and mental health challenges (17.6%, with Hispanic/Latino treatment gaps). Each factor was associated with 5–14 percentage point reductions in listing probability, comparable to clinical metrics. Psychosocial risk and SDOH factors explained 42.6% of racial disparities in listing decisions for Asian patients, exceeding liver health metrics (36.8%) and contributing to 94.6% collective explanation of differences. Priority interventions should target caregiver support, substance use, mental health, and patient education. This framework for systematically analyzing patient circumstances could enhance understanding of care decisions and health disparities.

## Introduction

Healthcare access and outcomes remain fundamentally shaped by social and economic circumstances^1,2^, but quantifying these relationships has proven challenging^3–11^. Nowhere is this more evident than in liver transplantation (LT), where scarce organs must be allocated based on both medical need and psychosocial stability^12–16^. Although the Model for End-Stage Liver Disease (MELD) score provides a standardized measure of medical urgency^17^, transplant decisions incorporate extensive psychosocial assessments that are often documented in unstructured clinical notes. These assessments capture crucial factors such as history of substance use and social support systems that directly influence transplant eligibility and outcomes^11,18–27^.

Although structured psychosocial assessment tools like the Stanford Integrated Psychosocial Assessment for Transplant (SIPAT) have validated the importance of social factors in transplant outcomes^22,23^, transplant centers vary in their use of the SIPAT and in their processes for systematically capturing patient life circumstances. Available structured SDOH measures are largely limited to neighborhood-level area deprivation indices and are inconsistently collected^3,4^, while individual-level patient circumstances are predominantly documented in unstructured clinical notes^5^.

Current transplant evaluation practices mandate documentation of these psychosocial factors^28,29^, but their unstructured nature has hindered large-scale analysis of how they influence care decisions, such as the addition of a candidate to the waiting list for transplantation. This limitation is particularly significant given the persistent disparities in transplant access across gender, race, and socioeconomic status^20,21,30–38^. Understanding how social factors shape transplant decisions requires methods to systematically analyze previously inaccessible information in clinical notes. Recent advances in natural language processing (NLP) and large language models (LLMs) have enabled systematic extraction of social and economic circumstances^9,39–41^, offering a promising approach to address these challenges.

We developed an artificial intelligence (AI) framework that extracts standardized representations of patient circumstances from transplant evaluation notes across 23 dimensions of social determinants using LLMs. Our analysis demonstrates four key findings. First, LLMs can reliably extract psychosocial risk and social determinants of health (SDOH) factors related to LT decisions as defined by clinicians and social workers. Second, we identify the factors that vary the most across different patient subgroups and with different policy changes over time. Third, patterns of adverse social factors vary systematically between demographic groups and explain observed disparities in listing decisions. Lastly, these psychosocial risk and SDOH “snapshots” substantially improve the prediction of progression through transplant evaluation when combined with clinical data.

We present a systematic analysis that demonstrates that previously unstructured information can reveal hidden patterns in how patient circumstances influence transplant decisions, while revealing persistent disparities that warrant attention. The primary objective of this study is to leverage systematically extracted psychosocial and social determinant factors to quantify their influence on liver transplant evaluation outcomes relative to clinical variables and explain demographic disparities through modifiable factors, enabling the identification of specific intervention targets. Our analysis covers over ten years of LT data from the University of California, San Francisco, a large academic transplant center that conducts over 200 liver transplants per year and serves patients across the Western United States, including California, Nevada, Oregon, Washington, and Hawaii. We quantified the effects of psychosocial risk factors and SDOH within subgroups based on race and ethnicity, as well as sex. Our assessment uncovers compelling new insights into how large-scale machine learning (ML) methods can illuminate the impact of psychosocial risk and SDOH factors on LT decisions, especially in the context of existing health disparities. Because psychosocial risk and SDOH comprise a significant number of modifiable factors related to health outcomes^42^, our work creates actionable insights to help clinicians and healthcare professionals address high-impact factors.

## Results

### Datasets and model training

**Fig. 1:**
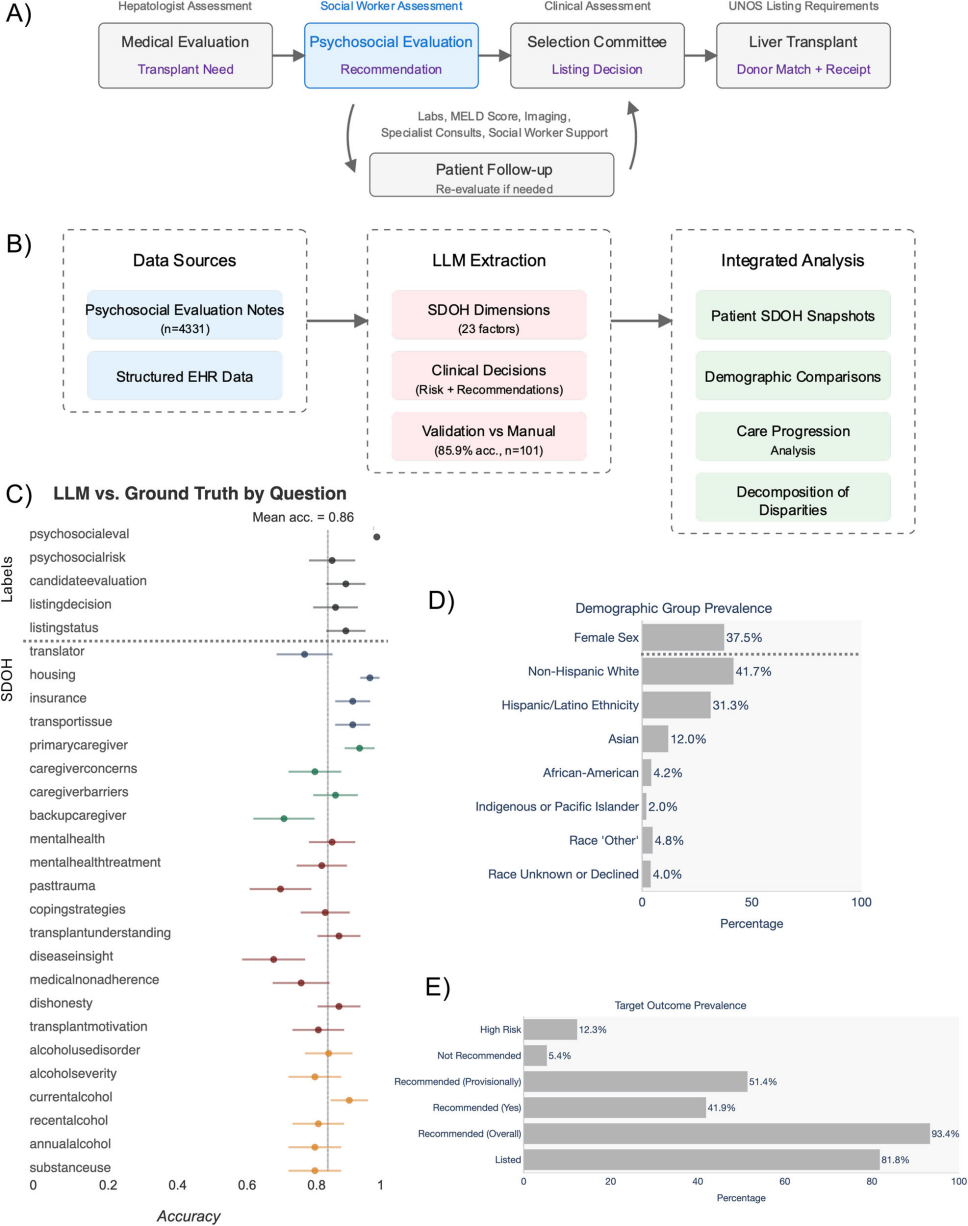
Framework for extracting and analyzing psychosocial risk and SDOH information from transplant evaluation notes. **A** Schematic overview of the liver transplant care journey. Decision outcomes shown in purple. **B** Schematic overview of psychosocial risk and SDOH snapshot creation and analysis pipeline. Clinical notes are processed using LLMs to extract both (i) 23 psychosocial risk and SDOH dimensions describing patient circumstances* and (ii) clinical decisions/outcomes not captured in structured data (e.g., psychosocial risk assessments, transplant recommendations). These extracted elements are combined with structured clinical and demographic data from the EHR to create comprehensive patient snapshots at evaluation. The integrated data enables (i) comparison of psychosocial risk and SDOH factor prevalence across demographic groups, (ii) identification of transition points where specific factors impact care progression, and (iii) decomposition analysis of how psychosocial risk and SDOH patterns and clinical factors explain demographic differences in care access. This approach surfaces both individual-level circumstances and population-level patterns that can guide resource allocation and policy decisions. **C** Accuracy of GPT4-Turbo-128k vs. ground truth annotations (*n* = 101) for 28 questions, including 23 psychosocial risk and SDOH-related dimensions. **D** Demographic composition of the study cohort (*n* = 3704). **E** Prevalence of key clinical outcomes, including psychosocial recommendation status (Rec) and liver transplant (LT) listing rates. *Psychosocial risk and SDOH colored by related theme (yellow = ‘Substance Use’; green = ‘Social Support’; blue = ‘Access’, and red = ‘Psychological’).

We conducted a retrospective, longitudinal analysis using deidentified electronic medical record (EMR) data collected at the University of California, San Francisco. Our patient cohort includes 4331 adult patients evaluated for liver transplantation (LT) between 2012 and 2023 and extracted psychosocial evaluation notes from these patients. The final cohort (*n* = 3704) included patients with complete demographic and clinical data. See the study diagram in Figs. 1 and S1 for the cohort selection diagram.

Our evaluated cohort is demographically diverse (Fig. 1D), and the majority of patients have the full set of structured clinical data (*n* = 3704). The distribution of patient race and ethnicity within the cohort is 42% Non-Hispanic White, 31% Hispanic or Latino, 13% Asian, 4% Black or African-American, 2% Indigenous and Pacific Islanders, 5% Other, and 3% “Unknown or Declined” (unknown race or undisclosed race), with a gender distribution of 37.5% female and 62.5% male. Within this cohort, 41% of the patients have a diagnosis of hepatocellular carcinoma (HCC), with a higher prevalence for Asian patients (56.8%), and a lower prevalence than the average for female and Hispanic/Latino patients (30.1% and 37.6% respectively). Controlling for HCC exceptions, there are no statistically significant differences in liver disease severity between demographics at a population level, as estimated by MELD scores (Supplementary Fig. S3).

We analyze two clinical decisions in the liver transplant care journey (Fig. 1A). Each potential LT patient is assessed for psychosocial factors, documented in the psychosocial evaluation. Our first clinical decision is the recommendation of the psychosocial evaluation. After a psychosocial recommendation is given, the selection committee combines the recommendation with other clinical assessments to decide whether or not to list this LT patient with the United Network of Organ Sharing (UNOS). Our second clinical decision is the LT listing decision. Due to the many complexities in the transplant matching process, we focused on the immediate effects of the decision of the LT panel compared to the more complex outcomes related to the actual completion of the transplant. The detailed prevalence rates for transplant listing by demographic group for all evaluated patients are shown in Table S1 and Supplementary Fig. S2.

To extract psychosocial risk and SDOH factors, we employed a HIPAA-compliant LLM (gpt-4-turbo128k). To assess the benefits of factors on predictive performance, we trained an Extreme Gradient Boosting (XGBoost) model. To understand and quantify the effects of psychosocial risk and SDOH on the LT decision-making process, we examined whether the addition of these factors would improve the performance of a predictive model trained to predict LT outcomes. Specifically, for each patient evaluated for LT, we make two predictions: (1) whether they will be recommended for LT based on the psychosocial evaluation, and (2) whether they will be listed for LT. These two prediction models are developed using a combination of clinical, demographic, and extracted psychosocial risk and SDOH factors. We examined the interpretability of the predictive model using SHAP values^43^, which indicate the features that most impact the model predictions and the direction and strength of that impact.

### LLMs can extract liver transplant psychosocial risk and SDOH factors

Building on recent work that demonstrated that LLMs can extract SDOH factors^9^, we developed a large-scale extraction pipeline that could amplify the domain expertise of LT specialists. We defined 23 psychosocial risk and SDOH categories based on the recent literature and hospital policies^18,19^ in close collaboration with licensed clinical social workers and a transplant clinician. These categories include any history of substance use, access factors for the patient, social support, and mental health factors (Fig. 1C). The text description shown shows a few word summary of the factor derived from each question in the note query (see full list of questions in Supplementary Table S2; Supplementary Figs. S4 and S5 present confusion matrices for LLM-derived information.) Using our HIPAA-compliant LLM, we extracted these dimensions from psychosocial evaluation notes of patients considered for LT. The length of the notes ranged from 221 to 4972 tokens (mean: 1592, SD: 507). We created an “SDOH snapshot” for each patient, capturing key psychosocial risk and SDOH factors that can influence LT outcomes. The extraction accuracy was validated against 101 expert annotations from licensed clinical social workers and a transplant clinician.

When validated against manual annotations in a random subset (*n* = 101), our approach achieved an average accuracy of 0.859 across all psychosocial risk and SDOH categories (95% CI: 0.846–0.872). The accuracy for the individual categories ranged from 0.70 (95% CI: 0.61–0.79) for the disease insight of the patient to 0.98 (95% CI: 0.95–1.00) for housing instability (Fig. 1C). Our results demonstrate that LLMs can reliably extract LT psychosocial risk and SDOH snapshots comparable to human subject matter experts.

### Psychosocial risk and SDOH factors reveal differing patterns across patients

**Fig. 2:**
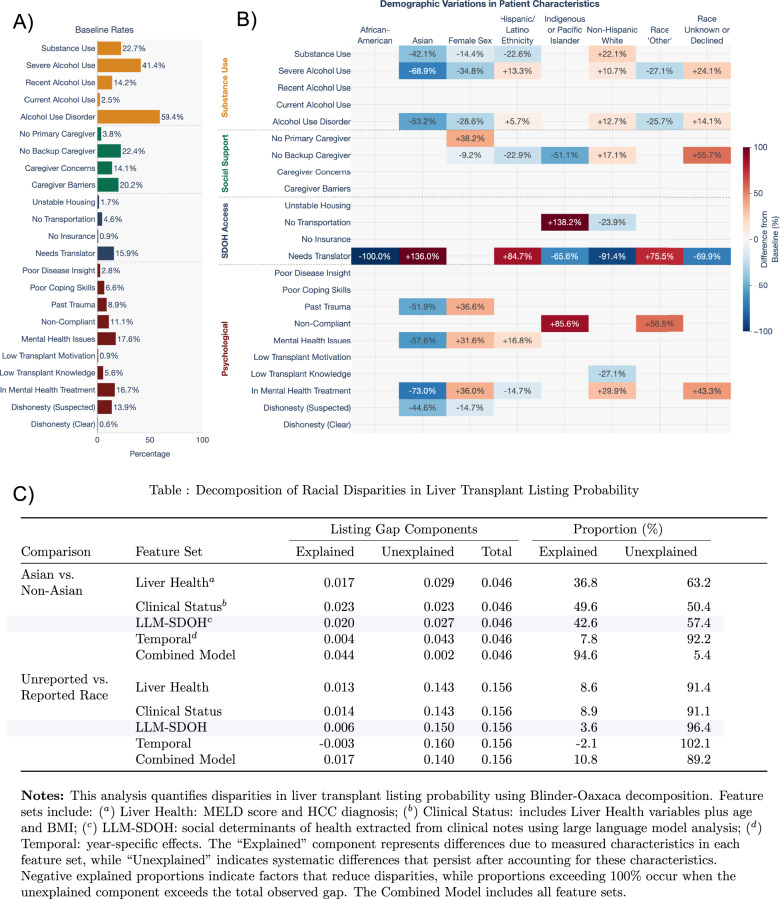
Analysis of demographic disparities in liver transplant listing rates. **A** Baseline prevalence rates for psychosocial and substance use factors identified in clinical notes. **B** Heatmap showing statistically significant differences in factor prevalence across demographic groups (two-proportion z-tests, *p* < 0.05, FDR-corrected), expressed as percentage-point differences from baseline; colored boxes represent statistically significant differences from patient average; blue indicates lower rates, red indicates higher rates. **C** Blinder-Oaxaca decomposition analysis quantifying explained and unexplained components of listing probability disparities, showing independent contributions of liver health metrics, psychosocial risk and SDOH features, and temporal effects.

We analyzed differences in psychosocial risk and SDOH snapshots across patient subgroups. Because not all patient data contains complete clinical information, this missingness and censorship can affect downstream analysis and models^6^. As a result, we studied patients with complete clinical information and patients with incomplete clinical information separately. Among patients with complete clinical data (*n* = 3704), we observed significant demographic variations in psychosocial Risk and SDOH factors (Fig. 2B, Supplementary Fig. S11). We focused initially on this subset to be able to control for the contribution of clinical factors and disease status when modeling psychosocial risk and SDOH impacts. Asian patients consistently demonstrated lower rates across multiple psychosocial domains compared to the mean baseline, including severe alcohol use (−68.9%) and mental health treatment (−73.0%). Gender analysis revealed female patients were 38.2% more likely to have no identified primary caregiver. Female patients also had higher rates of mental health issues (+31.6%) and ongoing treatment (+36.0%), and of past trauma (+36.6%), while showing lower rates of severe alcohol use (−35.8%). Hispanic or Latino patients exhibited higher rates of mental health issues (+16.8%) but lower reported rates of mental health treatment (−14.0%), suggesting a potential treatment gap of 31.5%. Indigenous/Pacific Islander patients showed higher rates in transportation access needs (+138.2%) and history of medical non-adherence (+85.6%). Non-Hispanic White patients showed higher rates of substance use (+22.1%) while experiencing lower rates of transportation issues (−23.9%). ‘Other’ race patients showed a higher prevalence of history of medical non-adherence (+56.5%) and translator need (+75.5%). Patients with unknown race or who declined to specify their race in UCSF tended to have higher rates of severe alcohol use (+24.1%), ongoing mental health treatment (+43.3%) and 55.7% lower rates of backup caregivers identified.

Some patients in our study lacked complete clinical data (*n* = 548), potentially due to greater vulnerability or early loss to follow-up. This group revealed generally higher adverse psychosocial risk and SDOH prevalence (Supplementary Fig. S7). To avoid overlooking important variations potentially associated with these more vulnerable patients, we then examined the larger cohort with demographic data (*n* = 4243) (Supplementary Fig. S6). While our previous findings of psychosocial risk and SDOH prevalence across demographics remained robust, several additional associations emerged. African-American patients showed higher rates of low transplant process knowledge (+70%) and non-alcohol substance use (+29.8%). Patients with undisclosed race demonstrated increased rates across multiple dimensions, including suspected dishonesty (+40.4%), poor coping skills (+87.2%), and were more likely to have caregivers about whom social workers expressed concerns regarding adequate support provision (+43.7%). The expanded analysis also revealed that Indigenous/Pacific Islander patients showed higher rates of severe alcohol use history (+29.0%). Female patients demonstrated a higher prevalence of poor coping skills (+21.2%) while maintaining all previously described psychosocial risk and SDOH factor associations. Transportation issues were significantly higher for patients of other (+74.6%) and undisclosed race (+67.2%).

Finally, focusing specifically on the subset of patients without full information (*n* = 548) revealed distinct patterns of psychosocial risk and SDOH prevalence. Social workers were more likely (+52.1%) to identify potential barriers to the primary caregiver’s ability to provide adequate support for patients of undisclosed race. Asian patients in this subset maintained a lower prevalence of substance use and psychosocial risk factors compared to other demographics, but showed notably higher translator needs than the overall cohort (+252.7%). Female patients in this group continued to show lower rates of alcohol use disorder (−19.7%) and prior severe alcohol use (−22.6%), while maintaining higher rates of past trauma (+43.0%), ongoing mental health challenges (+19.5%), and mental health treatment (+31.3%).

### Psychosocial risk and SDOH factors reveal temporal shifts

Temporal analysis revealed notable shifts in both demographics and psychosocial health factors over the study period, including an increase in the proportion of Latino patients from 22% in 2012 to 43% in 2023 (Fig. S8a) and rising rates of recent alcohol use (18% in 2012 to 28% in 2023) (Fig. S9a). Patients requiring translation services steadily increased from 14.5% to 20.8% over the same time period (Fig. S9b). These increases potentially reflect transplant policy changes at UCSF and epidemiological shifts in liver disease burden. The observed temporal increase in documented mental health factors like trauma (11% in 2012 to 18% in 2023) and mental health challenges (24% in 2012 to 36% in 2023) may reflect evolving screening practices and reduced stigma rather than true trends in prevalence. Leveraging the snapshots in this way, we can see both recent ‘shocks’ (the jump in the prevalence of reported ongoing mental health issues from 2022 to 2023) and longer-term trends in the case of the reporting of past trauma, translator requirements, and the increase in the proportion of Hispanic or Latino patients evaluated for transplant at UCSF.

### SDOH factors explain racial disparities

**Fig. 3:**
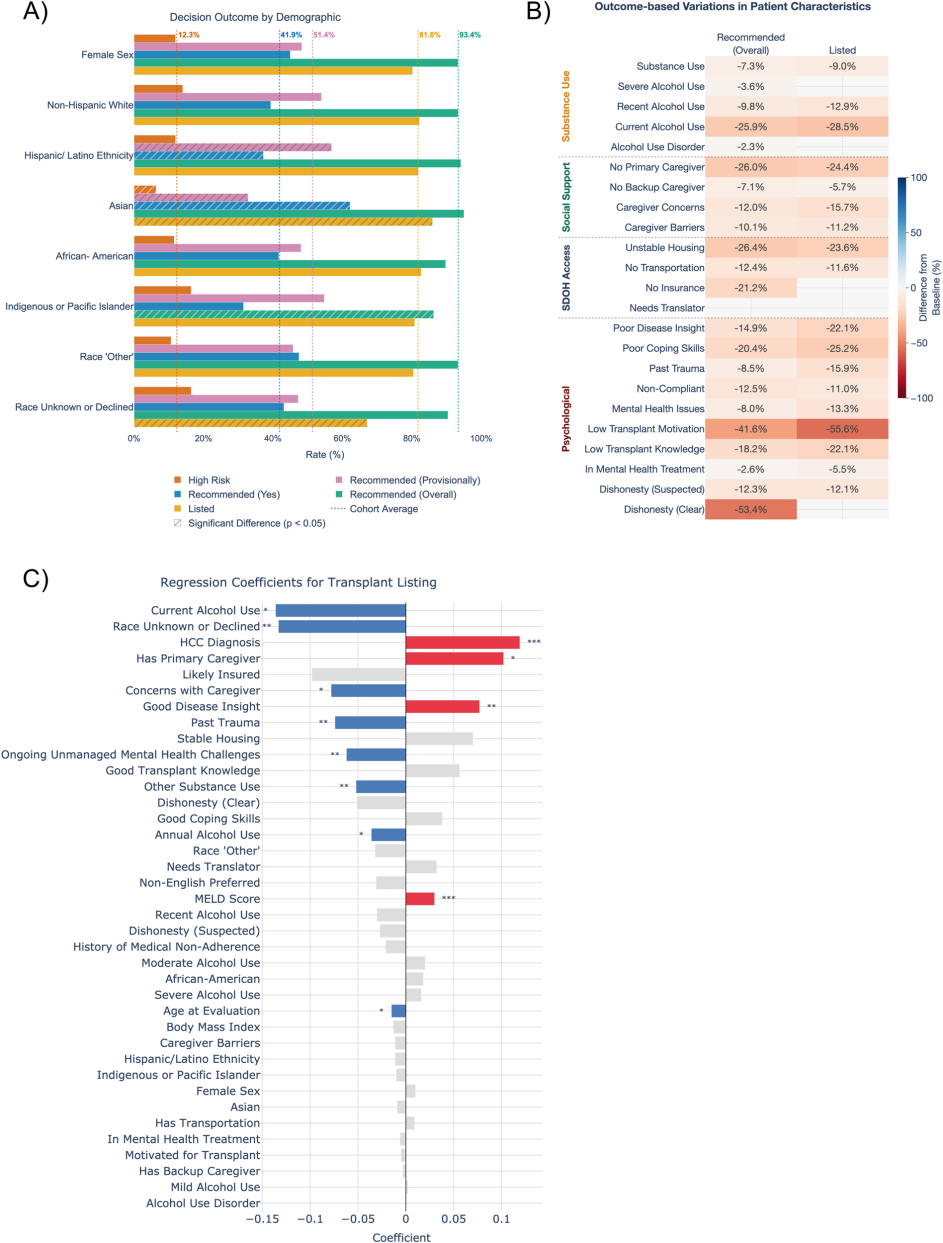
Demographic and psychosocial risk/SDOH variation across liver transplant outcomes. **A** Percentage of patients reaching each evaluation milestone^†^ stratified by demographic group, showing progression from initial psychosocial risk assessment through listing. Striped bars indicate significant differences from overall cohort means (FDR-corrected two-proportion z-tests). **B** Heatmap showing significant differences in psychosocial risk and SDOH factor prevalence between patients who did versus did not achieve each outcome (two-proportion z-tests, *p* < 0.05, FDR-corrected); blue indicates higher rates, red indicates lower rates, blank cells indicate non-significant differences. **C** OLS regression coefficients with LLM-derived, clinical, and demographic features. Significant coefficients marked (*p < 0.05, **p < 0.01, ***p < 0.001) and colored based on whether they have a positive (red) or negative (blue) impact on listing. ^†^Note on outcome classifications: “Recommended (Yes)” refers only to patients receiving unconditional recommendations, while “Recommended (Provisionally)” is a separate group. These two recommendation types are mutually exclusive. The “Overall” group includes both provisionally and unconditionally recommended patients. All other outcomes can co-occur.

Racial disparities in LT are well-documented, but the mechanisms underlying these differences remain poorly understood. We hypothesized that psychosocial risk and SDOH factors might explain a substantial portion of these disparities. In our dataset, Asian patients showed significantly higher listing rates while patients with unknown or undisclosed race showed markedly lower rates compared to the patient average, making these populations particularly informative to understanding disparities (Fig. 3A). To rigorously quantify the how much of these differences could be explained by measurable factors, including psychosocial risk and SDOH, we employed the Blinder-Oaxaca decomposition method–a statistical technique that quantifies how much of an outcome gap between groups can be attributed to differences in measured characteristics versus other unexplained factors (Fig. 2C, see table notes for variables included in each model). For Asian patients, psychosocial risk and SDOH factors in isolation explained 42.6% of listing outcome gaps—more than measures of liver health, which explained 36.8%. Combined features explained 94.6% of variance in Asian patient listing outcomes. However, for patients with unreported race that is either unknown or undisclosed, only 10.8% of listing outcome gaps were explained by combined features (8.6% liver health, 3.6% psychosocial risk and SDOH).

While the Blinder-Oaxaca analysis revealed how much of the racial disparities could be explained by measured factors, we next sought to understand which specific psychosocial risk factors and SDOH had the strongest influence on listing decisions. To investigate this, we identify the factors most negatively associated with recommendation and listing (Fig. 3B), with listed patients less likely to have low transplant motivation (−55.6%), current alcohol use (−28.5%), poor coping skills (−25.2%), and unstable housing (−23.6%). They are also less likely to lack a primary caregiver (−24.4%). To further examine the relative individual contributions of clinical, demographic, and psychosocial risk and SDOH factors to listing likelihood, we carry out regression analysis (Fig. 3C). We find that when controlling for clinical factors and psychosocial risk and SDOH factors, some racial differences were no longer statistically significant. However, other racial information, such as having an unknown or undisclosed race, greatly decreased the rate of LT listing (−0.13, *p* < 0.001), further indicating possible disparities unexplained by factors drawn from the psychosocial evaluation for these patients. As expected, clinical factors such as hepatocellular carcinoma (HCC) (0.12, *p* < 0.001) and MELD score (0.03, *p* < 0.001) have significant positive impact on transplant listing; however, some psychosocial risk and SDOH factors such as good disease insight (0.08, *p* < 0.001) also have a positive impact on listing above and beyond a standard deviation change in MELD score. Psychosocial risk and SDOH factors with negative effects on LT listing outcome include current alcohol use (−0.14, *p* < 0.05), concerns with the primary caregiver (−0.08, *p* < 0.01), past trauma (−0.07, *p* < 0.01), ongoing unmanaged mental health challenges (−0.06, *p* < 0.01), and other non-alcohol substance use (−0.05, *p* < 0.01). Our analysis reveals important patterns in how adverse social determinants of health cluster together (Fig. S10). Patients with alcohol use disorder or severe substance use histories frequently face multiple concurrent social challenges. The co-occurrence analysis particularly highlights the relationship between active mental health challenges and other adverse factors, including past trauma, limited coping skills, and housing instability. We also observe clustering among factors related to medical understanding—specifically, patients with limited disease insight often also demonstrate poor coping skills and insufficient transplant knowledge (Fig. S10).

### Psychosocial risk and SDOH factors improve prediction for LT psychosocial recommendation and listing

**Fig. 4:**
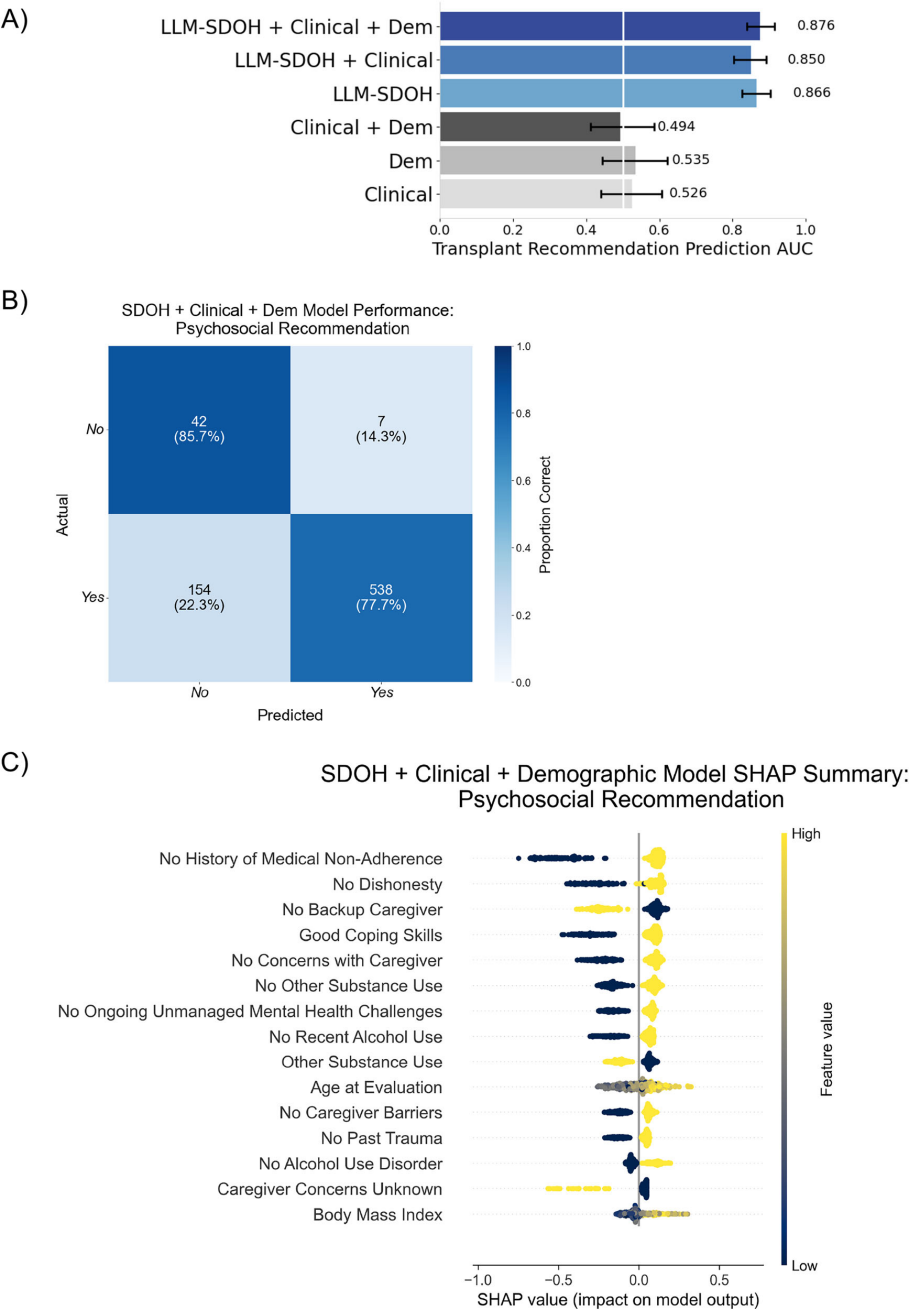
Model performance and feature analysis for psychosocial recommendation prediction. **A** Comparison of average AUROC (w. 95% CI) across six combinations of clinical, demographic, and LLM-derived feature sets. Feature sets including LLM-derived features shown in blue. **B** Confusion matrix for the LLM-SDOH + Clinical + Demographic combined feature model with normalized percentages over true values (rows). **C** SHAP (SHapley Additive exPlanations) values for the top 15 features for the model with all feature sets.

**Fig. 5:**
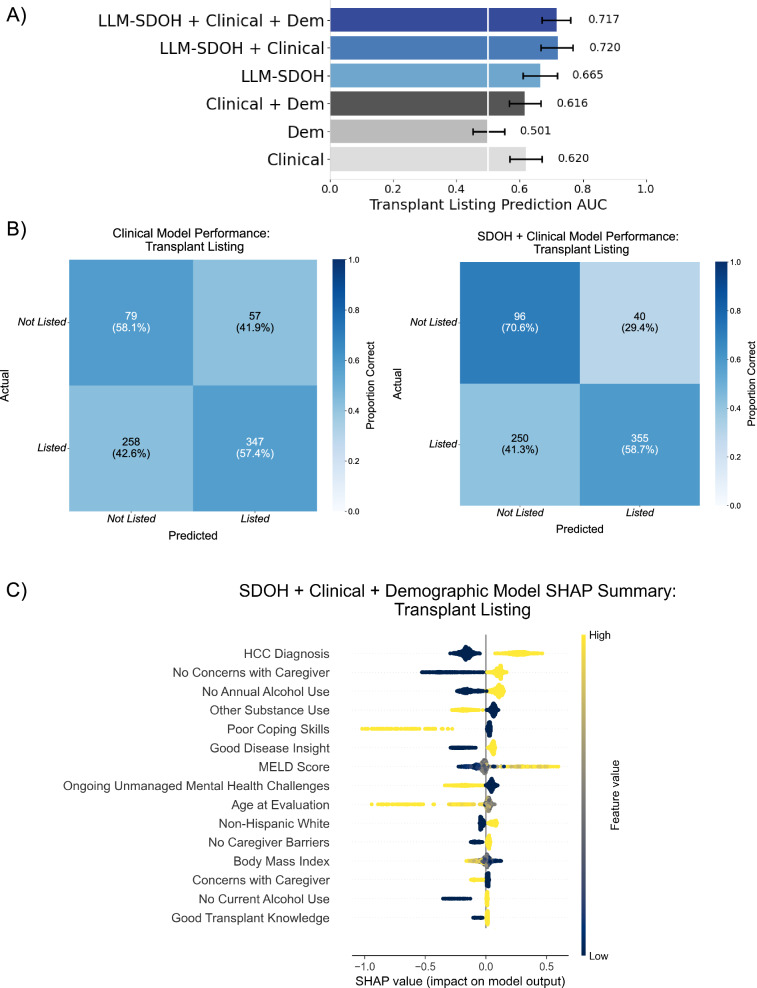
Model performance and feature analysis for liver transplant listing prediction. **A** Comparison of average AUROC (w. 95% CI) across six combinations of clinical, demographic, and LLM-derived feature sets. Feature sets including LLM-derived features shown in blue. **B** Confusion matrix for the Clinical (left) and Clinical + LLM-SDOH combined feature model (right) with normalized percentages over true values (rows). **C** SHAP (SHapley Additive exPlanations) values for the top 15 features for the model with all feature sets.

Recognizing that comparisons across demographic groups do not give a complete picture of the influence of psychosocial risk and SDOH factors on individual patient outcomes, we leverage non-linear predictive modeling to provide a more individualized understanding of how such characteristics can influence the prediction of LT decisions. We demonstrated that integration of LLM-derived psychosocial risk and SDOH features with demographic information and clinical covariates, including MELD score, HCC status and BMI, significantly improved outcome prediction. For psychosocial recommendation, the area under the receiver-operator curve (AUROC) increased 77.3% from 0.494 (95% CI: 0.413–0.585) to 0.876 (95% CI: 0.8380.915) (Fig. 4). For eventual successful listing, AUROC improved 16.4% from 0.616 (95% CI: 0.566–0.666) to 0.717 (95% CI: 0.670–0.762) (Fig. 5).

Among patients recommended based on their psychosocial evaluations, models predicting successful listing achieved an AUROC of 0.589 (95% CI: 0.534–0.643) using clinical features alone (Fig. 4). Including psychosocial risk and SDOH factors increased AUROC to 0.680 (95% CI: 0.628–0.732)—a 15.5% improvement. Psychosocial risk and SDOH-only models achieved an AUROC of 0.641 (95% CI: 0.5860.696), exceeding clinical-feature models and suggesting these factors may better predict listing outcomes than traditional clinical metrics, including measures of liver health (i.e., MELD score, HCC status) and other characteristics known to influence the likelihood of transplant (e.g., BMI and age). These results indicate that psychosocial risk and SDOH factor impact extends beyond initial recommendations and likely captures patient characteristics distinct from clinical measures.

**Fig. 6:**
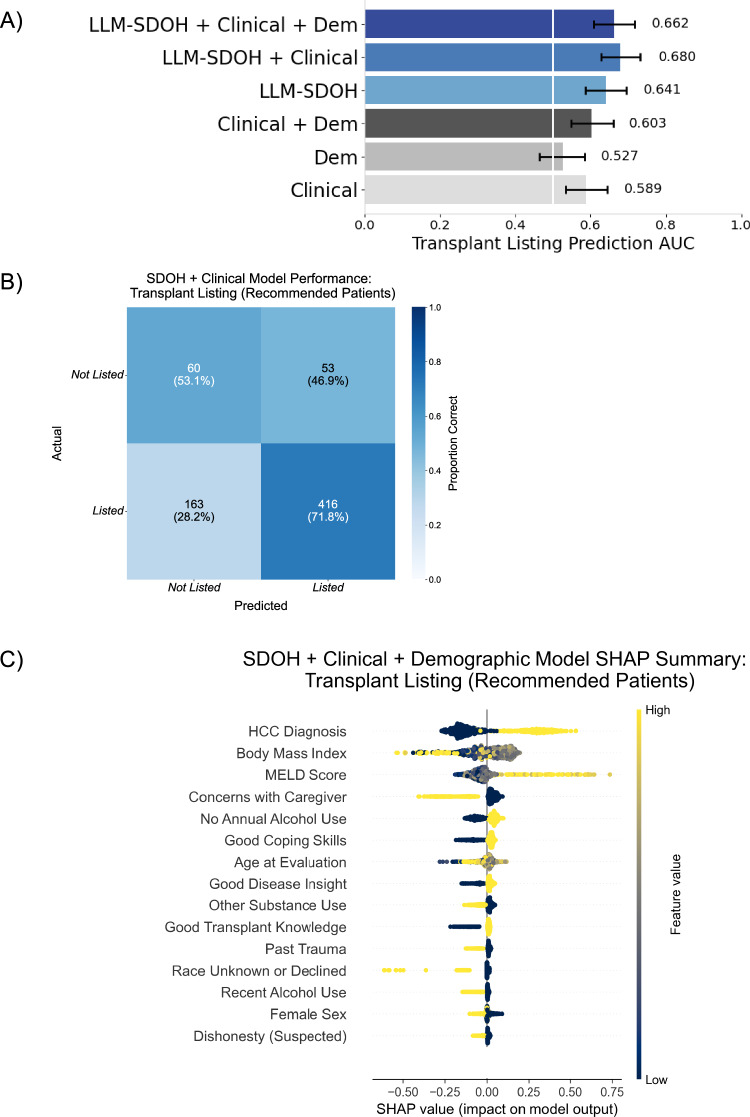
Model performance and feature analysis for liver transplant listing prediction in patients with psychosocial recommendations. **A** Comparison of average AUROC (w. 95% CI) across six combinations of clinical, demographic, and LLM-derived feature sets. Feature sets including LLM-derived features shown in blue. **B** Confusion matrix for the Clinical + LLM-SDOH combined feature model with normalized percentages over true values (rows). **C** SHAP (SHapley Additive exPlanations) values for the^20^ top 15 features for the model with all feature sets.

Shapley Additive exPlanations (SHAP) analysis was carried out to help identify key predictors across the transplant process and assess the relative importance of clinical and psychosocial risk and SDOH features (Fig. 6). For psychosocial recommendation, top predictors were lack of medical non-adherence, lack of caregiver concerns or dishonesty, good coping skills, and absence of non-alcohol substance use. Successful listing was positively associated with HCC diagnosis, MELD score, a lack of caregiver concerns, no alcohol use in the past year, and good disease insight, while substance use, poor coping skills, ongoing mental health challenges, and higher age had a negative impact. Sensitivity analyses confirmed model robustness: excluding patients with listing decisions documented in evaluation notes maintained stable performance (AUROC 0.719 vs. 0.717), and including patients with incomplete clinical data showed consistent results across feature sets (Supplementary Figs. S12, S13).

We benchmark how LLM-derived psychosocial risk and SDOH factors compare to NLP baseline models such as Bag-of-Words (BOW) and clinical Text Analysis and Knowledge Extraction System (cTAKES) concepts^44^. We assessed two binary outcomes: psychosocial recommendation (93% base rate) and eventual successful listing (81% base rate) (Fig. 1D). Models using cTAKES features performed the worst (e.g., psychosocial recommendation AUROC of 0.52; 95% CI 0.44–0.60). Models using LLM-derived and BOW features performed higher (Supplementary Fig. S14, Table S3). The model using BOW features outperformed the model using LLM features: for psychosocial recommendation, the model using BOW features has an AUROC of 0.91 (95% CI: 0.86–0.95), whereas the LLM features have an AUROC of 0.87 with 95% CI: 0.82–0.90. However, we note that the LLM features are far more interpretable and seem to avoid the label leakage that likely contributes to the performance of the models based on BOW features (Supplementary Fig. S15).

## Discussion

Our findings demonstrate that systematic extraction of psychosocial risk and SDOH information from clinical notes improves understanding of liver transplant access and outcomes. Through our analysis, we show that LLMs can reliably extract patient circumstances from unstructured documentation, achieving 0.70–0.98 accuracy across multiple psychosocial risk and SDOH dimensions (Fig. 1). This capability proves valuable given our finding that psychosocial risk and SDOH-only models outperform clinical-feature models in predicting successful listing, indicating these factors capture unique and nonredundant information about patient progression through the transplant evaluation process (Figs. 5, 6). Our analysis identifies key psychosocial risk and SDOH factors contributing to both LT decisions, including current or recent alcohol use, lack of social support (e.g., primary caregiver), unstable housing and medical-literacy-associated factors such as disease insight, coping skills, and transplant knowledge (Figs. 3–6). These dimensions also help explain racial disparities in our cohort, and highlight remaining unexplained disparities for follow-up (Fig. 2). We emphasize that the intent of our modeling is to understand the effects of psychosocial risk and SDOH on liver transplant outcomes and decisions, not to provide an AI tool to reproduce those decisions. Beyond our quantitative results, our work has several generalizable insights and larger implications for the medical community.

First, our work directly ties machine learning approaches to psychosocial risk and SDOH factors relevant to LT decision-making that can provide guidance for healthcare practitioners. As larger medical datasets and more sophisticated LLMs become available to medical researchers^9,10,45^, it is very likely that other liver transplantation centers may find slightly different patterns and biases depending on their patient population and policy decisions; however, our framework enables the understanding of specific psychosocial risk and SDOH factors that significantly affect outcomes at different stages.

The lack of social support emerges as a major barrier in our UCSF cohort. Specifically, the absence of primary caregivers is associated with reduced progression through psychosocial recommendation and LT listing, with caregiver-related factors emerging as significant predictors of outcomes across models. Medical literacy and psychological resilience represent another critical barrier. Key predictive factors include disease insight, coping skills, history of medical non-adherence, and ongoing mental health challenges. These findings point to several potential interventions: structured caregiver support programs including financial compensation, expanded psychoeducation through multilingual recorded sessions, and enhanced peer support through transplant mentors and support groups. Our temporal analysis further reveals increasing documentation of translator needs, alcohol use, and mental health concerns, suggesting growing demands for integrated translation, substance use, and mental health services. While our findings reveal important disparities among patients who reach transplant evaluation, addressing inequities will require interventions at multiple levels: improving initial access to transplant referral, enhancing support during evaluation (our focus), and optimizing post-transplant care.

The demographic patterns in our analysis point to specific opportunities for intervention. In our patient cohort, Indigenous/Pacific Islander patients face higher rates of transportation barriers and lower initial recommendation rates, suggesting a need for enhanced support services during early evaluation stages. Hispanic or Latino patients show higher rates of mental health issues but lower rates of ongoing treatment, while being more likely to receive provisional recommendations. This pattern indicates potential benefits from integrated, culturally-sensitive mental health support combined with transplant evaluation follow-up. These findings become apparent when examining our complete patient cohort, showing how analyses restricted to patients with complete clinical data may underestimate both the prevalence and impact of certain psychosocial risk and SDOH factors. In order to model the range of interventions available, our binary classification task of recommendations could be expanded to make distinctions between provisional and full recommendations for care. Further, our analysis focuses on patients already referred for transplant evaluation, representing a selected population that has navigated initial healthcare access barriers. We may underestimate disparities, as patients facing the most severe barriers may never reach transplant evaluation. Future work should examine the factors affecting pre-referral access to transplant centers.

Second, our framework of developing standardized psychosocial risk and SDOH representation to model the effects of social and economic circumstances could be used to model a wide range of medical applications—including other organ transplantation decisions, maternal health outcomes, and care for chronic conditions. The rapid proliferation of LLMs used on clinical data^46^ has demonstrated tremendous potential to improve health equity. Although researchers have raised concerns about the potential for ML models and particularly LLMs to amplify existing disparities^47^, we believe that careful construction and validation of models could enable a new wave of understanding the interplay of both human and ML bias to improve health outcomes.

Third, we highlight that our LLM extraction of psychosocial risk and SDOH factors relies on an assumed ground truth when validating these factors. Our analysis implicitly sets social worker labels as the gold standard; however, more scrupulous analysis of automated labeling by LLMs may be warranted, especially since automated NLP labeling techniques may skew downstream ML results for different patient subpopulations. In order to better understand the full LT psychosocial evaluation process, the transcript of interviews could be studied, potentially with the help of automated NLP tools.

Lastly, although EHR data and newly unlocked unstructured notes can shed more insight into the process, we must be vigilant for documentation biases that can magnify the “streetlight effect”—the tendency to search for answers only where data is easily available^48^. While SDOH factors explain a substantial portion of listing outcome gaps for Asian patients, significant unexplained differences persist for other groups, particularly patients with undisclosed race. This variation in explanatory power points to a key analytical challenge: those most vulnerable to early loss from the transplant evaluation process often lack complete clinical data, potentially excluding them from traditional analyses. The forces that drive health outcomes extend far beyond clinical encounters, and while we expand analyzable information beyond traditional structured clinical data, we remain limited by what providers choose to document.

In summary, our study has several important limitations. Our single-center design may limit generalizability, though our diverse patient population and comprehensive temporal analysis provide valuable insights. We rely on clinical documentation that may contain systematic biases in how psychosocial risk factors and SDOH are recorded or assessed across different patient populations^49^. If documentation practices vary by demographics—whether through differential surveillance, implicit bias, or varying levels of detail—LLM extraction could amplify these institutional inequities rather than revealing true clinical risk patterns, as highlighted in recent reviews of AI bias in healthcare^47,50^. Our validation framework assumes that social worker assessments in clinical notes represent an accurate reflection of patients’ actual social circumstances, when in reality these assessments may themselves be influenced by clinical judgment, patient disclosure patterns, or documentation constraints^51^. Finally, we cannot definitively distinguish whether observed demographic differences reflect genuine disparities in social circumstances versus differential documentation practices^52^.

These limitations underscore the critical importance of careful validation and interpretation when using AI-extracted social determinants to understand healthcare disparities. However, they also highlight the potential for this systematic approach to reveal patterns previously hidden in unstructured data. As machine learning technologies advance and medical datasets expand, thoughtful application of these methods offers unprecedented opportunities to understand how patient circumstances shape health outcomes and to develop more equitable, evidence-based interventions.

## Methods

### Data and preprocessing

We analyzed psychosocial evaluation notes from 4331 adult patients evaluated for liver transplantation (LT) at a large academic medical center between 2012 and 2023. The final cohort (*n* = 3704) included patients with complete demographic and clinical data. The cohort’s race-ethnicity distribution was 42% Non-Hispanic White, 31% Hispanic or Latino, 13% Asian, 4% Black or African-American, 2% Indigenous and Pacific Islanders, 5% Other, and 3% Unknown or Declined, with a gender distribution of 37.46% female and 62.54% male. Numerical variables were normalized using scikit-learn’s StandardScaler^53^. Categorical variables were one-hot encoded. For Bag-of-Words (BOW) baseline comparison models, we used NLTK^54^ for text preprocessing and scikit-learn for feature extraction and selection.

The UCSF IRB approval number is “Study# 23-40630.” Consent of participants was waived by the IRB. For the patient who did proceed to transplantation, the transplanted organs were allocated by the United Network for Organ Sharing, which is the non-profit organization that manages the Organ Procurement and Transplantation Network (OPTN) under contract with the United States government, and were not procured from prisoners.

### Psychosocial risk and SDOH factor definition and extraction

We defined 23 psychosocial risk and Social Determinants of Health (SDOH) categories based on recent literature^18,19,22^ and hospital policies. These categories included substance use history, patient access factors, social support, and mental health factors. The categorization was developed in close collaboration with licensed clinical social workers and a transplant clinician. We employed a privacy-preserving version of GPT-4-Turbo-128k to survey these dimensions from clinical notes, creating a “Psychosocial Risk and SDOH snapshot” for each patient, capturing key factors that may influence LT outcomes. Extraction accuracy was validated against 101 expert annotations.

### Model development

XGBoost^55^ models were developed to predict two key outcomes: psychosocial recommendation and eventual successful listing. We used 80% (*n* = 2963) of the data for training and hyperparameter tuning and 20% (*n* = 741) for testing, with stratification by outcome. Models were created using: (1) Clinical covariates only, (2) Clinical covariates + LLM-derived Psychosocial risk and SDOH features, and (3) Clinical covariates + Psychosocial risk and SDOH features + demographic factors. Downsampling of the majority class was performed using RandomUnderSampler^56^. Hyperparameter tuning used grid search with 5-fold cross-validation, exploring: max depth^3,6,9^, learning rate [0.01, 0.1, 0.2], n estimators [100, 300, 500], subsample and colsample bytree [0.7, 0.8, 0.9], and gamma [0, 0.1, 0.2]. Ordinary Least Squares (OLS) models were created with the linear model function from the statsmodels Python package^57^ with ‘HCV3’ robust standard errors.

### Evaluation

Model performance was evaluated using the area under the receiver-operator curve (AUROC), sensitivity, and specificity on the held-out test set. SHAP values were used to interpret feature importance^43^. To further analyze differences in outcomes across demographic groups, we employed linear probability models and Blinder-Oaxaca decomposition^58–60^, both implemented with the Python statsmodels package^57^. Blinder-Oaxaca decomposition quantifies outcome gaps between demographic groups by separating differences attributable to measured characteristics (explained component) from systematic differences that persist after accounting for these characteristics (unexplained component). We implemented this analysis using statsmodels with linear probability models, testing feature sets independently and in combination. Feature sets included: Liver Health (MELD score, HCC diagnosis); Clinical Status (Liver Health plus age and BMI); LLM-SDOH (social determinant and psychosocial risk factors across four domains as detailed in Table S4); and Temporal (year of evaluation). The Combined Model incorporated all feature sets simultaneously to identify which factors most contribute to demographic disparities in listing decisions.

### Statistical analysis of psychosocial risk and SDOH by demographic

We conducted systematic analyses of psychosocial and clinical factors across demographic groups for the set of patients with all clinical features, including admissions information (*n* = 3695), using a structured statistical approach implemented in Python. For each factor, we calculated baseline prevalence rates and demographic-specific variations using two-proportion z-tests with 95% confidence intervals, implemented through scipy.stats^61^. Statistical testing employed the chi2 contingency and norm.cdf functions from scipy.stats for chi-square tests and z-score calculations, respectively. Multiple comparison adjustment was performed using the multipletests function from statsmodels.stats.multitest^57^ with the Benjamini–Hochberg procedure to control false discovery rate across demographic comparisons. All statistical tests were conducted with *α* = 0.05, and results were stratified by factor domains (Social Support, Access, Psychological, and Substance Use) to enable domain-specific evaluation of demographic patterns. Temporal trends of prevalence across psychosocial risk and SDOH factors and demographics were visualized with line graphs.

### Psychosocial risk and SDOH co-occurrence

Co-occurrence patterns between adverse psychosocial risk and SDOH factors were analyzed using a normalized matrix approach. For each pair of binary factors *i* and *j*, we calculated the percentage of cases where factor *j* was present given the presence of factor *i*, yielding an asymmetric co-occurrence matrix. The computation was performed using matrix multiplication of binary indicators, with normalization by factor prevalence to obtain conditional percentages. Each cell (*i,j*) represents the percentage of patients with factor *j* among those who had factor *i*, calculated as (*n*_*ij*_*/n*_*i*_) × 100, where *n*_*ij*_ is the count of patients with both factors and *n*_*i*_ is the count with factor *i*. The diagonal represents 100% by definition. Results were visualized as a heatmap to highlight co-occurrence patterns, revealing potential compound vulnerabilities in the patient population.

## Supplementary information

Supplementary Information

## Data Availability

The clinical datasets analyzed in this study contain de-identified clinical information, portions of which could be shared based on reasonable request and in compliance with UCSF institutional policy.

## Code availability

Code for the modeling and analyses will be made available on GitHub.
