# Supplementary material for: A large language model-based approach to quantifying the effects of social determinants in liver transplant decisions
Source: NPJ Digit Med. 2025 Nov 17;8:665. doi: 10.1038/s41746-025-02025-y (PMC12623799; doi:10.1038/s41746-025-02025-y)
Supplement: Supplementary file 1 — Supplementary Information [file 41746_2025_2025_MOESM1_ESM.pdf]

Supplementary information

Study Context

Large Language Models (LLMs): Advanced AI systems that can read and understand clinical notes, similar to how an experienced physician reviews charts to extract key patient information. We used GPT-4-Turbo-128k, a state-of-the-art LLM, to systematically extract social and psychosocial factors from transplant evaluation notes that would typically require manual chart review by multiple clinicians.

Social Determinants Feature Extraction: The process of systematically identifying and categorizing patient characteristics from clinical documentation. Rather than reading each note individually, we used automated methods to consistently extract 23 social determinant factors across all 4,000+ patients. These categories were developed based on transplant literature and expert consultation with licensed clinical social workers and transplant clinicians, covering substance use history, social support systems, access barriers, and mental health factors.

Predictive Modeling: Computer algorithms that analyze patterns in large datasets to make predictions about patient outcomes. We used XGBoost, a widely-used machine learning approach, to identify which social factors most strongly predict transplant outcomes and to understand how these factors interact with traditional clinical measures like MELD scores. Models were trained using 80% of data with 20% held out for testing. To address class imbalance in outcomes (psychosocial recommendation: 93% base rate; transplant listing: 81% base rate), we applied RandomUnderSampler to downsample majority classes during model training. Our goal was not to replace clinical judgment, but to understand which documented social factors most strongly influence transplant progression.

Model Performance Assessment:

- **AUROC (Area Under Receiver-Operator Curve)**: A statistical measure of how well a predictive model performs, ranging from 0.5 (no better than random chance) to 1.0 (perfect prediction). Values above 0.7 are considered clinically useful; values above 0.8 indicate strong predictive ability.
- **SHAP Values**: A method to identify which factors most influence model predictions, helping us understand which social determinants matter most for transplant decisions. This shows the "weight" each factor carries in the decision-making process.

Disparities Analysis (Blinder-Oaxaca Decomposition): A statistical technique that quantifies how much of an outcome difference between demographic groups can be explained by measurable factors (like disease severity measures or psychosocial risk and SDOH) versus other unmeasured influences. This helps us understand what portion of disparities we can potentially address through targeted interventions.

Statistical Methods: Demographic comparisons used two-proportion z-tests with correction for multiple comparisons. Regression analyses employed standard statistical approaches with robust standard errors to account for data variability. Temporal trends were assessed using linear regression.

Validation: The process of testing model accuracy by comparing AI-extracted information against expert manual review. We validated our approach using 101 notes reviewed by licensed clinical social workers and transplant clinicians, achieving 85.9% average accuracy across psychosocial risk and SDOH categories.

A Supplementary Figures

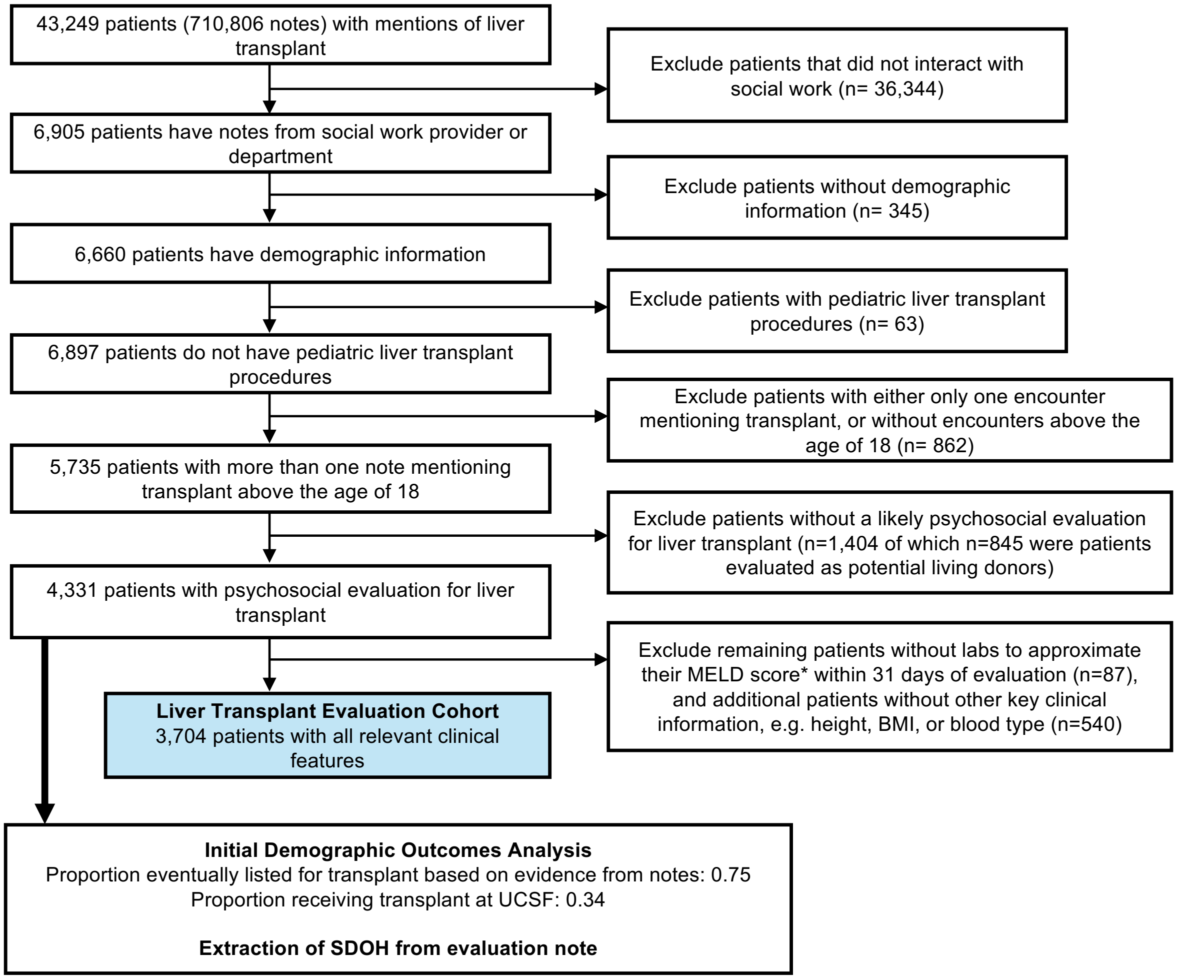

Figure S1: **Cohort Selection Process for Liver Transplant Evaluation Study**. This flowchart illustrates the step-by-step selection of patients for our LT study cohort. Starting with 43,249 patients (710,806 notes) mentioning liver transplant, we applied multiple exclusion criteria based on social work interaction, demographic factors, age, encounter frequency, presence of psychosocial evaluations, and availability of clinical data. The final cohort comprised 3,704 patients with complete psychosocial evaluations, demographic factors, and clinical data, including MELD scores within 31 days of evaluation. Proportions for listing and transplant receipt at UCSF are provided for the cohort with psychosocial evaluation notes.

Table S1: Demographic Characteristics and Listing Outcomes for Evaluated Patients

Characteristic	Listed No (n=1066)	Listed Yes (n=3265)	P-value
Race-Ethnicity			
Asian	100 (9.4)	414 (12.7)	
Black/African American	48 (4.5)	137 (4.2)	
Hispanic/Latino	313 (29.4)	1023 (31.3)	<0.001
Indigenous/Pacific	17 (1.6)	66 (2.0)	
Non-Hispanic White	428 (40.2)	1361 (41.7)	
Other	63 (5.9)	156 (4.8)	
Unknown/Declined	97 (9.1)	108 (3.3)	
Sex			
Female	469 (44.0)	1223 (37.5)	<0.001
Male	597 (56.0)	2042 (62.5)	

Note: Data presented as n (%). P-values calculated using chi-squared tests.

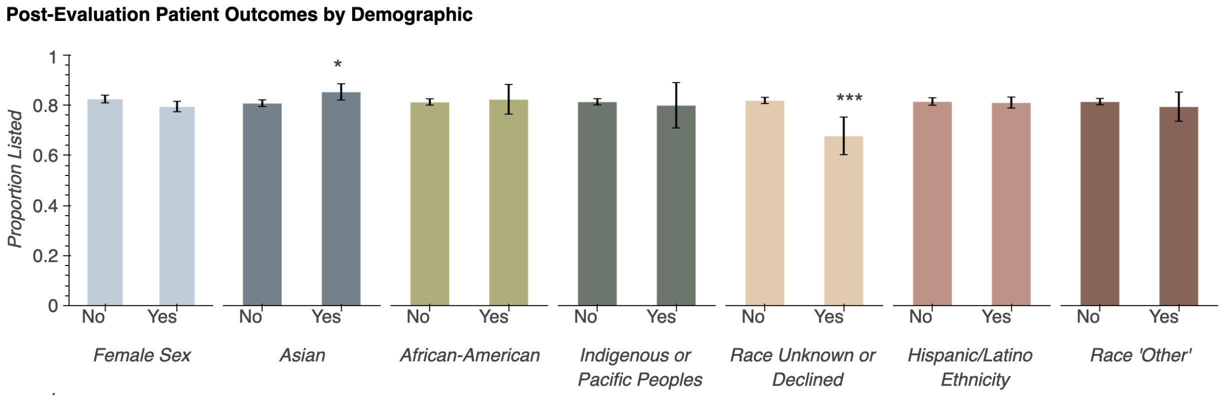

Figure S2: **Proportions of Patients Listed by Demographic Subgroup**. This figure displays the proportions of evaluated patients with full clinical data (n=3,704) listed for liver transplant across demographic subgroups. Error bars represent 95% confidence intervals. Statistical significance, determined using proportions z-tests, is denoted by asterisks (* $p<0.05$, ** $p<0.01$, *** $p<0.001$).

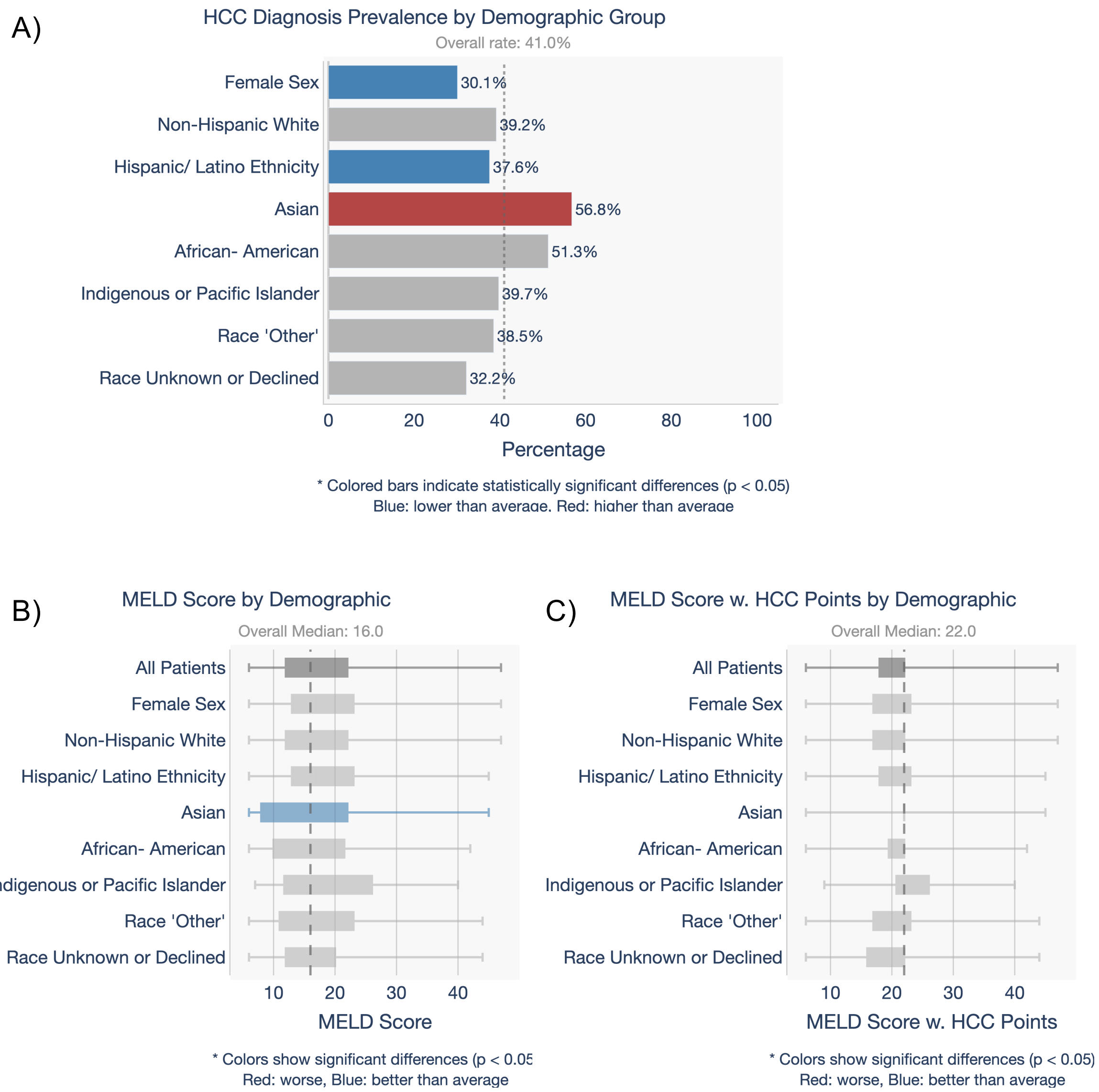

Figure S3: **Demographic distribution of liver disease severity metrics.** a) Hepatocellular carcinoma (HCC) prevalence across demographic groups. Colored bars indicate statistically significant differences from overall cohort mean (Fisher's exact test, $p<0.05$, FDR-corrected); blue indicates lower prevalence, red indicates higher prevalence. b) Distribution of laboratory MELD scores by demographic group. c) Distribution of MELD scores including HCC exception points (standardized to minimum score of 22 for HCC patients) by demographic group. For (b) and (c), box plots show median, interquartile range, and whiskers at 1.5× IQR; significance assessed by Kruskal-Wallis H test.

A.1 LLM-based Extraction from Clinical Notes: Detailed Methods

We present details on our LLM-based information extraction strategy including the prompt and the expert-informed questions and categories provided to the LLM in Table S2.

A.1.1 LLM Prompt

Prompt tuning with multiple system and task prompts was carried out on a small selection (n=20) of expert-labeled notes disjoint from the evaluation set. Following this process, the following prompt was used to instruct the GPT-4-Turbo-128k model for information extraction from clinical notes:

> *Assume the role of an expert medical professional. Your task is to extract and interpret vital information from clinical notes accurately. You will be provided with a clinical note, enclosed by triple backticks, and a set of questions. For each question related to the notes, choose the most accurate category (label) based on the evidence in the note. Format your analysis as JSON with 'Question Number' and 'Label'. If no evidence supports a category, return "Question Number": [number], "Label": "No evidence". Ensure precision in label identification and documentation. If multiple categories apply, select the most relevant one and justify your choice briefly. For the last two questions where no category choices are specified, answer each question in 50 words or less based on the note content and return that answer as the "Label".*

Categories returned with "No Label", "NA", or "No Evidence" were mapped to "Unknown." Following the mapping of categories, features were created by one-hot encoding the unique question category pairs for each note. Figures S4 and S5 present confusion matrices for LLM-derived information.

Table S2: Domain Expert-Informed Annotation Questions for LLM Query of Psychosocial Evaluation Notes

#	Question [Categories]
1	Does the note specifically provide a psychosocial evaluation addressing the patient's suitability for a liver transplant? [*Yes, No, Unknown*]
2	Does the patient require an English-language interpreter or translator? [*Yes, No, Unknown*]
3	What is the patient's housing situation? [*Stable Housing, Difficulty Paying for Housing, Without Housing (Undomiciled), Unknown*]
4	Does the patient have a designated caregiver? [*Yes, No, Unknown*]
5	Are there documented concerns about the caregiver's ability to provide the necessary care and support? [*Yes, No, Unknown*]
6	What possible barriers exist regarding the caregiver's ability to provide the necessary care and support? [*Health and Physical Capacity, Emotional and Mental Wellbeing, Employment or other Time or Financial Constraints, No Known Barriers, Unknown*]
7	Does the patient have a designated backup caregiver, also referred to as a secondary caregiver, or is there more than one caregiver identified who can take over if the primary caregiver is unable to fulfill their responsibilities? [*Yes, No, Unknown*]
8	Does the patient have any mental health issues that are actively affecting their daily functioning? [*Yes, No, Unknown*]
9	Is the patient actively receiving treatment, such as medications or therapy, for mental health issues? [*Yes, No, Unknown*]
10	Does the patient report any past trauma or abuse that remains unresolved, affecting their current well-being? [*Yes, No, Unknown*]
11	Does the patient's note show any documented evidence of past alcohol abuse or dependency that qualifies as addiction? [*Yes, No, Unknown*]
12	What was the severity of the patient's past alcohol use based on the documentation in the note? [*None, Mild, Moderate, Severe, Unknown*]
13	Is the patient currently using alcohol? [*Yes, No, Unknown*]
14	Has the patient used alcohol in the past 6 months? [*Yes, No, Unknown*]
15	Has the patient used alcohol in the past year? [*Yes, No, Unknown*]
16	Has the patient used any substances such as tobacco, marijuana, illicit drugs, or opioids in the past 6 months that raises health or treatment concerns? [*Yes, No, Unknown*]
17	Does the patient have healthy coping strategies to manage stress and challenges related to their medical condition? [*Yes, No, Unknown*]
18	Does the patient demonstrate a clear understanding of the requirements, procedures, and expected outcomes of the transplantation process? [*Yes, No, Unknown*]
19	Does the patient have insight into the causes of their liver disease and the reasons why they need a liver transplant? [*Yes, No, Unknown*]
20	Does the patient have a history of medical non-compliance (including failure to take medications as prescribed)? [*Yes, No, Unknown*]
21	According to the evidence in the note, was the patient dishonest or misleading during the evaluation? [*Yes, Suspected, No, Unknown*]
22	Does the patient have adequate health insurance coverage? [*Yes, No, Pending Confirmation, Unknown*]
23	Is the patient facing a transportation issue that would make it difficult to attend appointments? [*Distance/-Travel Time, Lack of Personal or Public Transportation, Financial Constraints, No Transportation Issues, Unknown*]
24	What is the patient's motivation for transplant? [*Highly Motivated, Somewhat Motivated, Not Motivated, Unknown*]
25	What is the overall psychosocial risk assigned to this candidate? [*Low, Moderate, High (Transplant Recommended), High (Transplant Not Recommended), Unknown*]
26	From a psychosocial perspective, is the patient recommended or considered a suitable candidate (e.g., reasonable, good, excellent) for a liver transplant? [*Recommended, Recommended Provided Compliance with Care Plan, Not Recommended, Unknown*]
27	Is there an addendum in the note with the listing decision? [*Yes, No, Unknown*]
28	What is the patient's transplant listing status, if it is mentioned in the note? [*Listed, Deferred, Declined/Denied, Status 1A, Temporarily Unfit, Unclear, Unknown*]
29	What specific risk factors or concerns have been reported that could impact the patient's suitability and fitness for a liver transplant? [*Open-ended*]
30	What specific protective factors have been reported that enhance the patient's suitability and fitness for a liver transplant? [*Open-ended*]

Figure S4: **Confusion matrices for LLM-derived information (Part 1)**

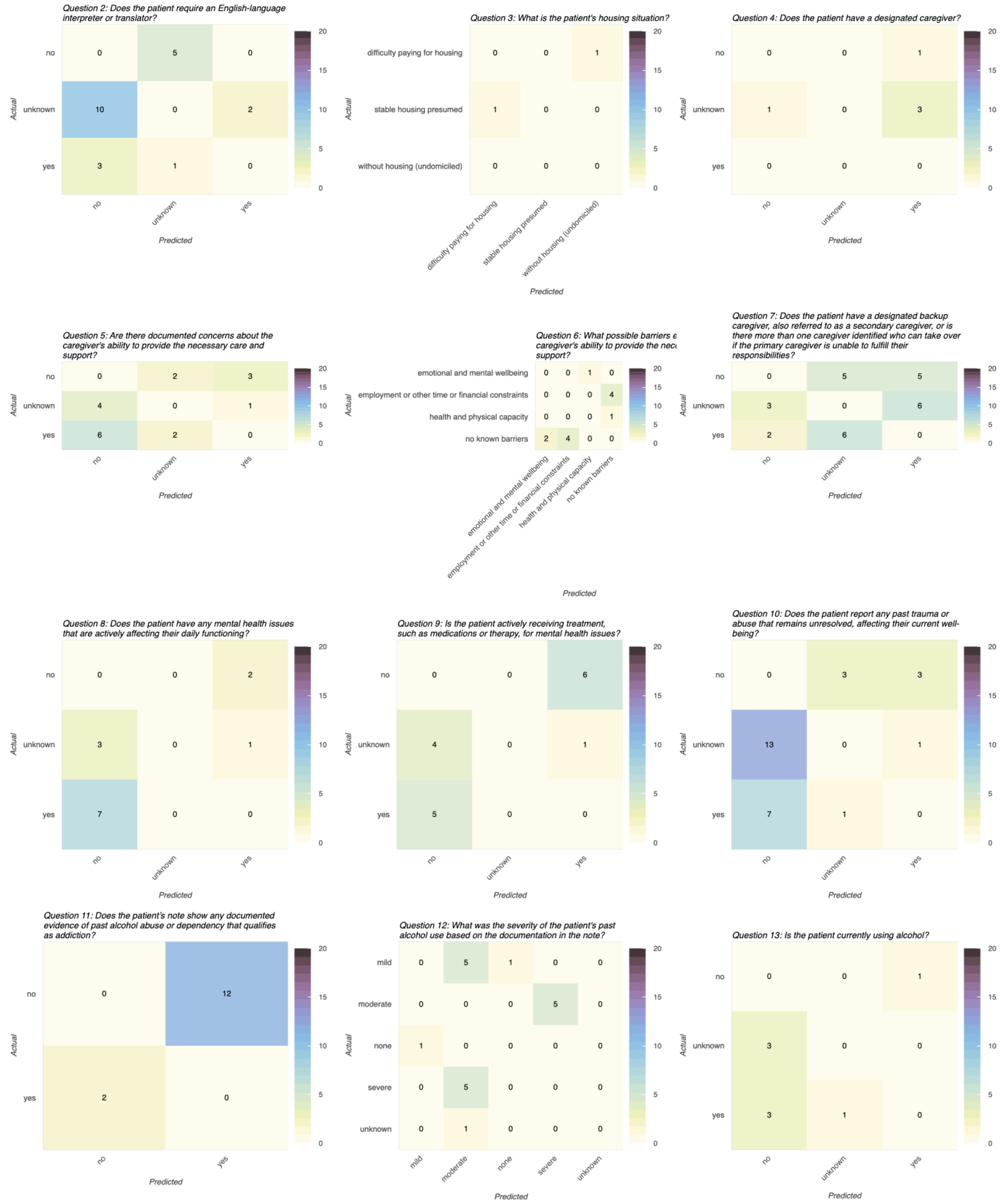

Figure S5: **Confusion matrices for LLM-derived information (Part 2)**

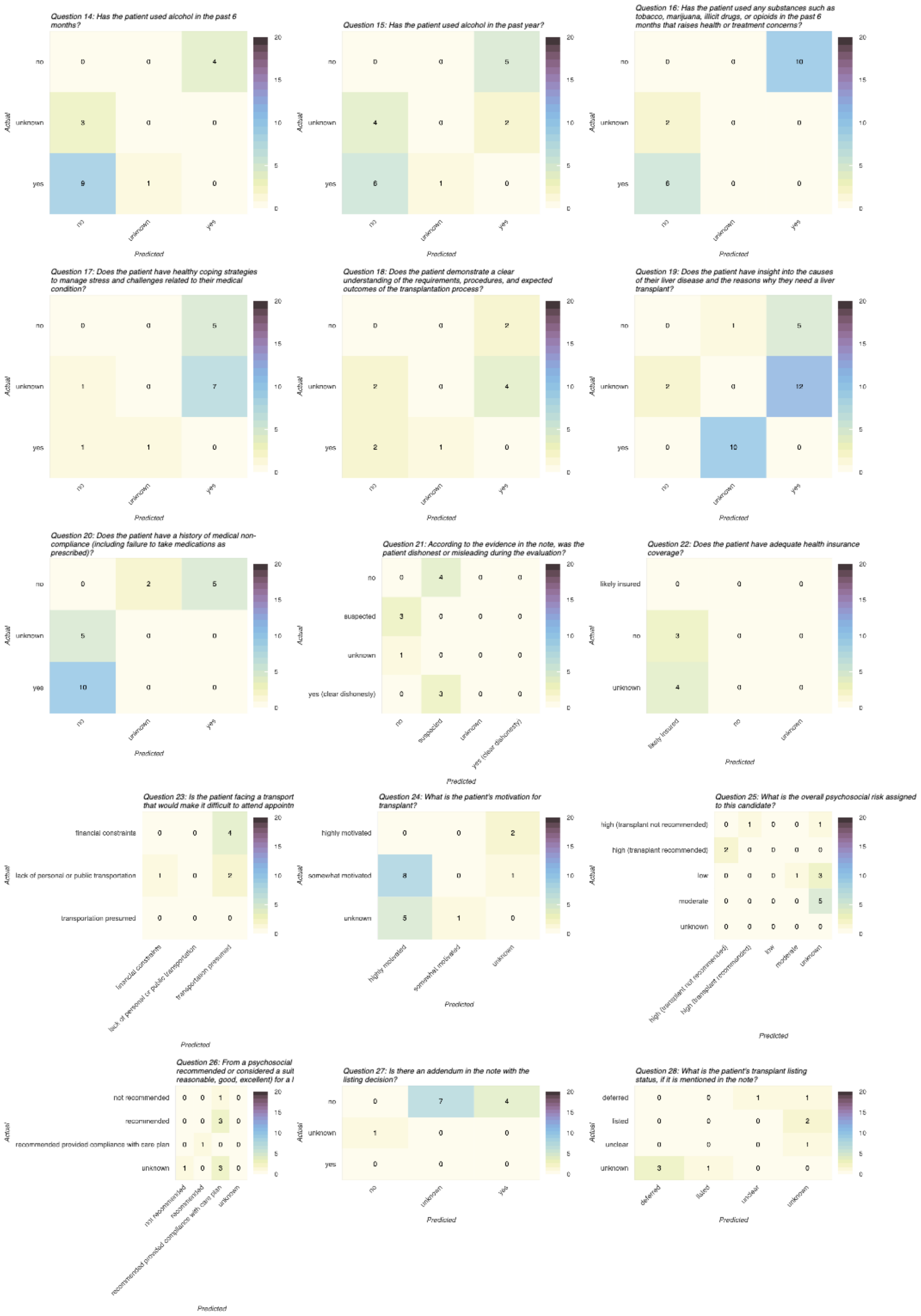

A.2 Psychosocial Risk and SDOH Factor Prevalence Expanded Results

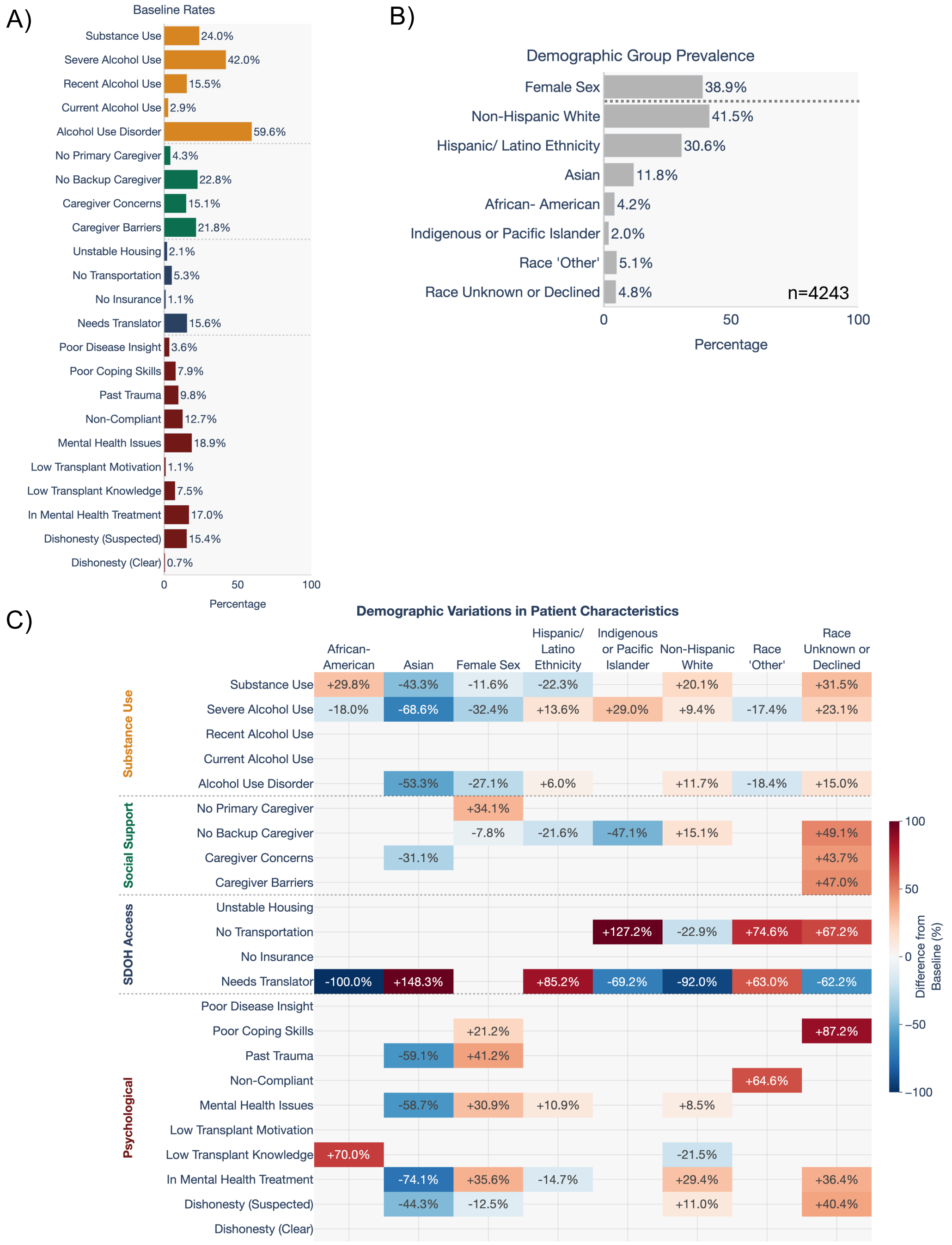

Figure S6: **Analysis of demographic disparities in liver transplant listing rates across all patients (n=4243).** a) Baseline prevalence rates for psychosocial and substance use factors identified in clinical notes. b) Demographic composition of the study cohort (n=4,243). c) Heat map of statistically significant differences in psychosocial risk and SDOH factor prevalence across patient demographics compared with the cohort average (two-proportion z-tests, $p < 0.05$, FDR-corrected); blue indicates higher rates, red indicates lower rates, blank cells indicate non-significant differences.

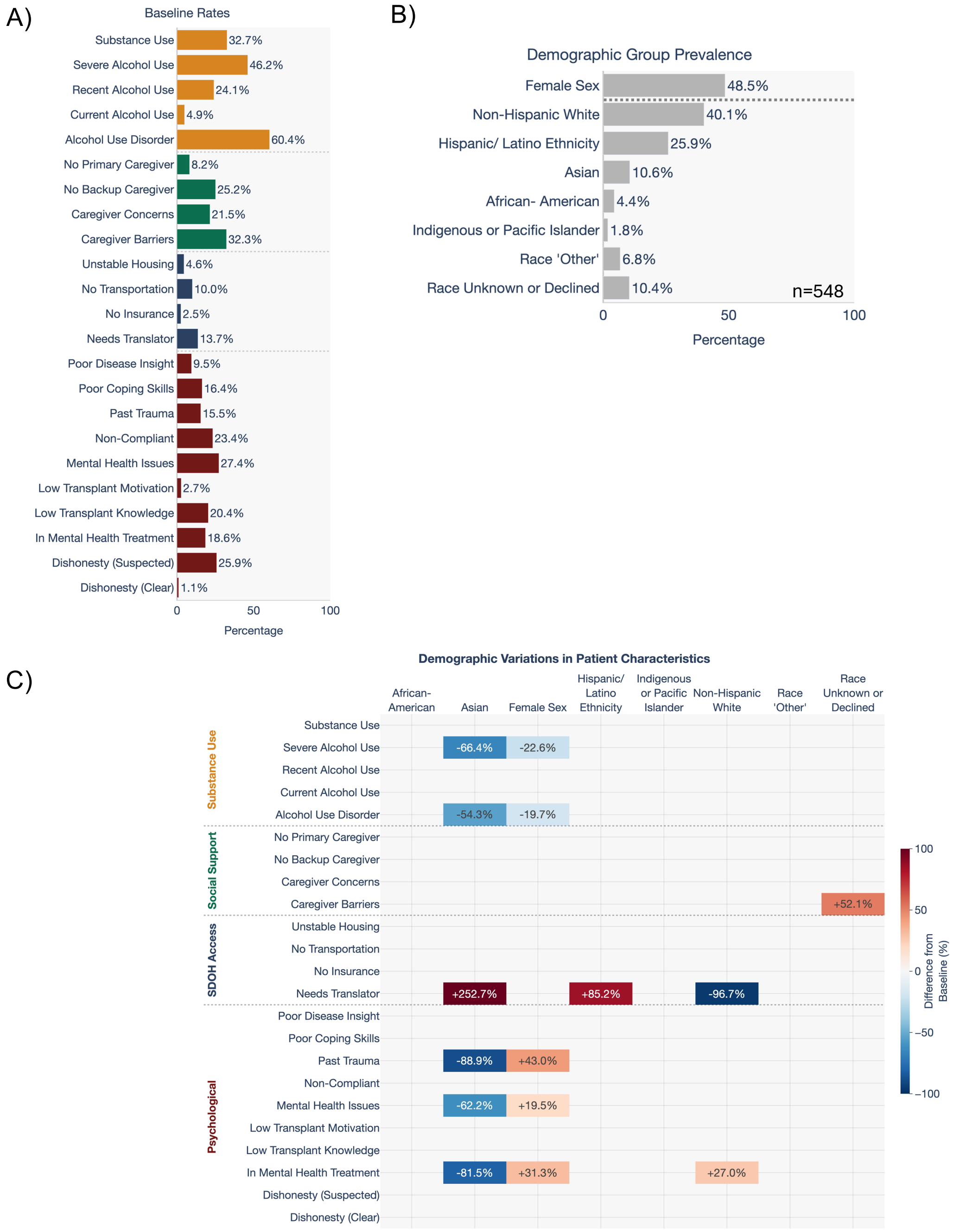

Figure S7: **Analysis of demographic disparities in liver transplant listing rates across patients missing data (n=548)** a) Baseline prevalence rates for psychosocial and substance use factors identified in clinical notes. b) Demographic composition of the study cohort (n=548). c) Heat map of statistically significant differences in psychosocial risk and SDOH factor prevalence across patient demographics compared with the cohort average (two-proportion z-tests, $p < 0.05$, FDR-corrected); blue indicates higher rates, red indicates lower rates, blank cells indicate non-significant differences.

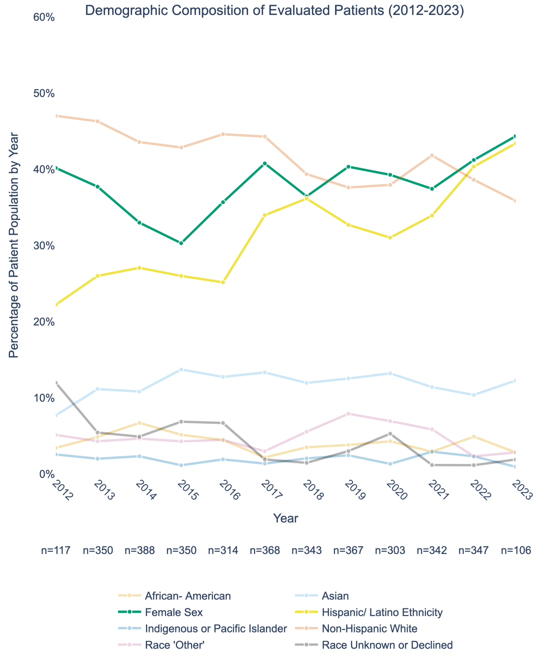

(a) Shifting demographics of patients evaluated for LT (2012-2023)

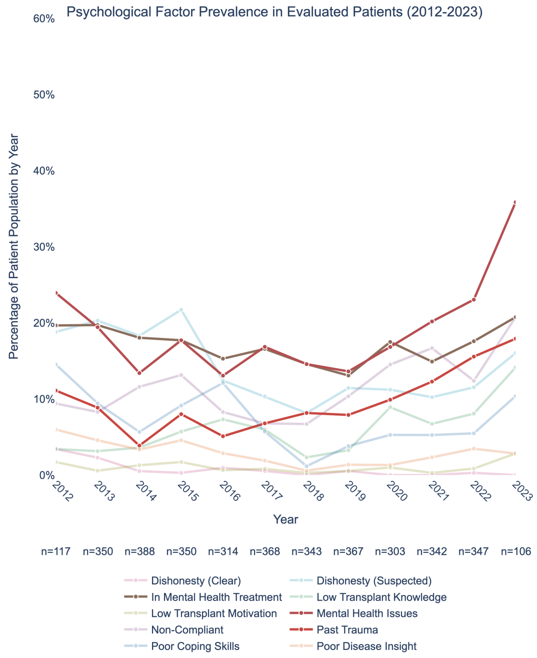

(b) Psychological profile trends in patients evaluated for LT (2012-2023)

Figure S8: **Temporal shifts in patient demographics and psychological factors for transplant evaluations (2012-2023).** (a) Trends in patient demographics over time (b) Trends in prevalence of assessed behavioral and mental health factors over time.

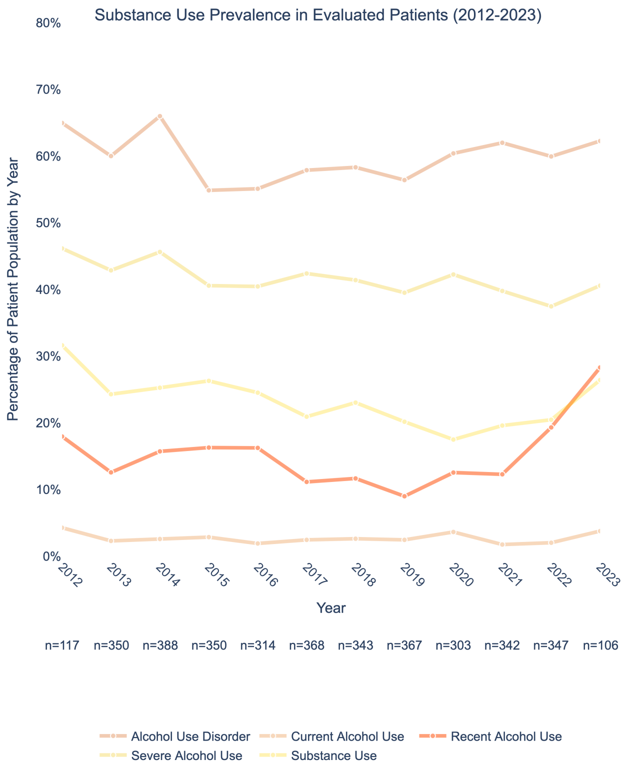

(a) Shifting substance us of patients evaluated for LT (2012-2023)

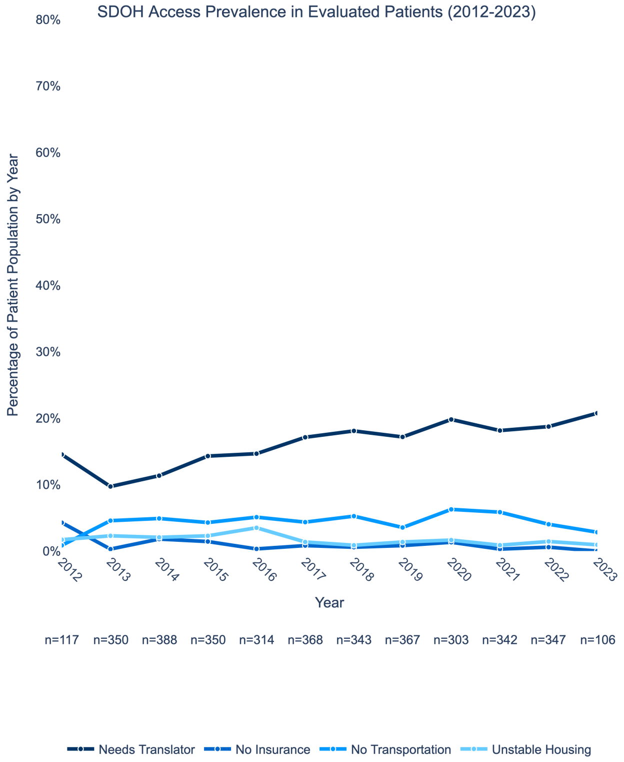

(b) Access-relevant trends in patients evaluated for LT (2012-2023)

Figure S9: **Temporal shifts in patient substance use and access-related psychosocial risk and SDOH factors for transplant evaluations (2012-2023).** (a) Trends in prevalence of patient substance use over time (b) Trends in prevalence of assessed access factors over time.

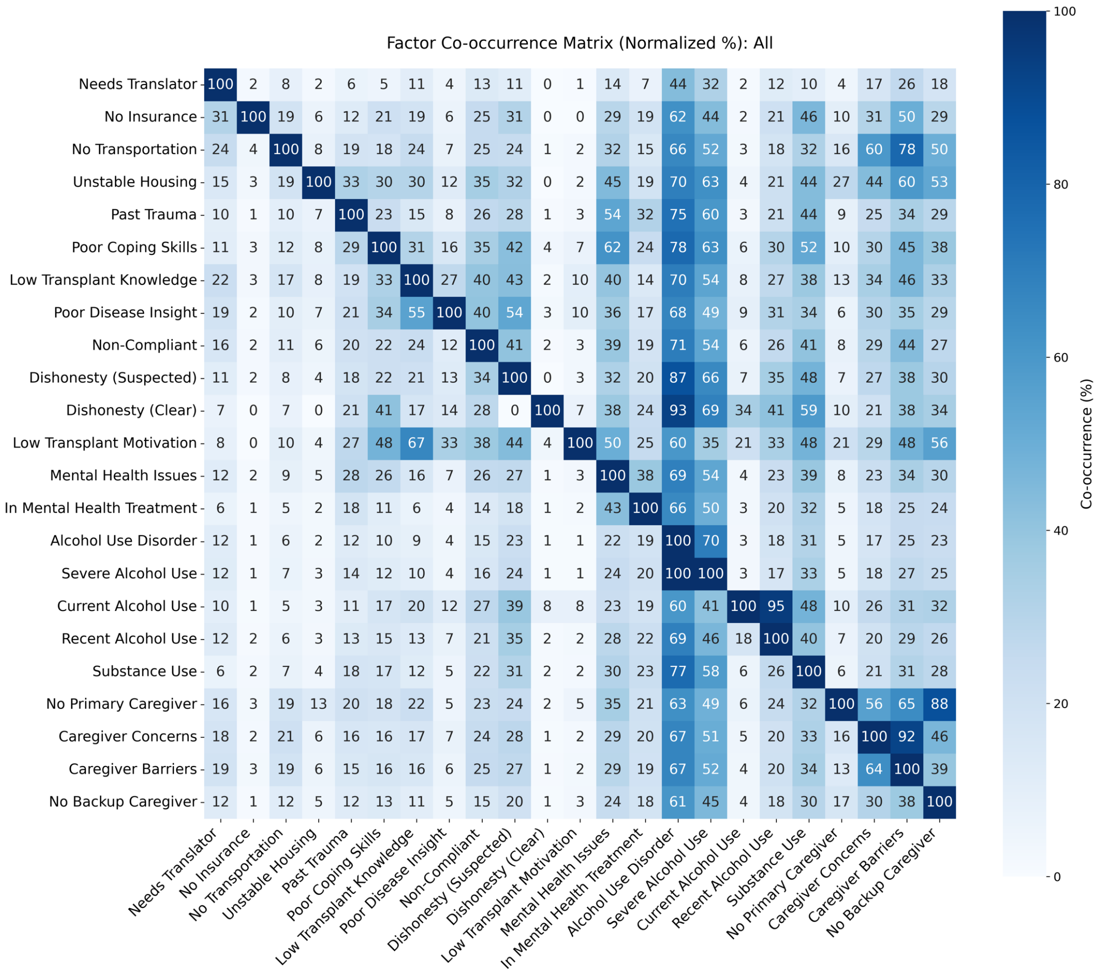

Figure S10: **Psychosocial risk and adverse SDOH co-occurrence matrix.** Heat map showing normalized pairwise co-occurrence of psychosocial risk and adverse SDOH factors across patients; darker blue indicates higher rates.

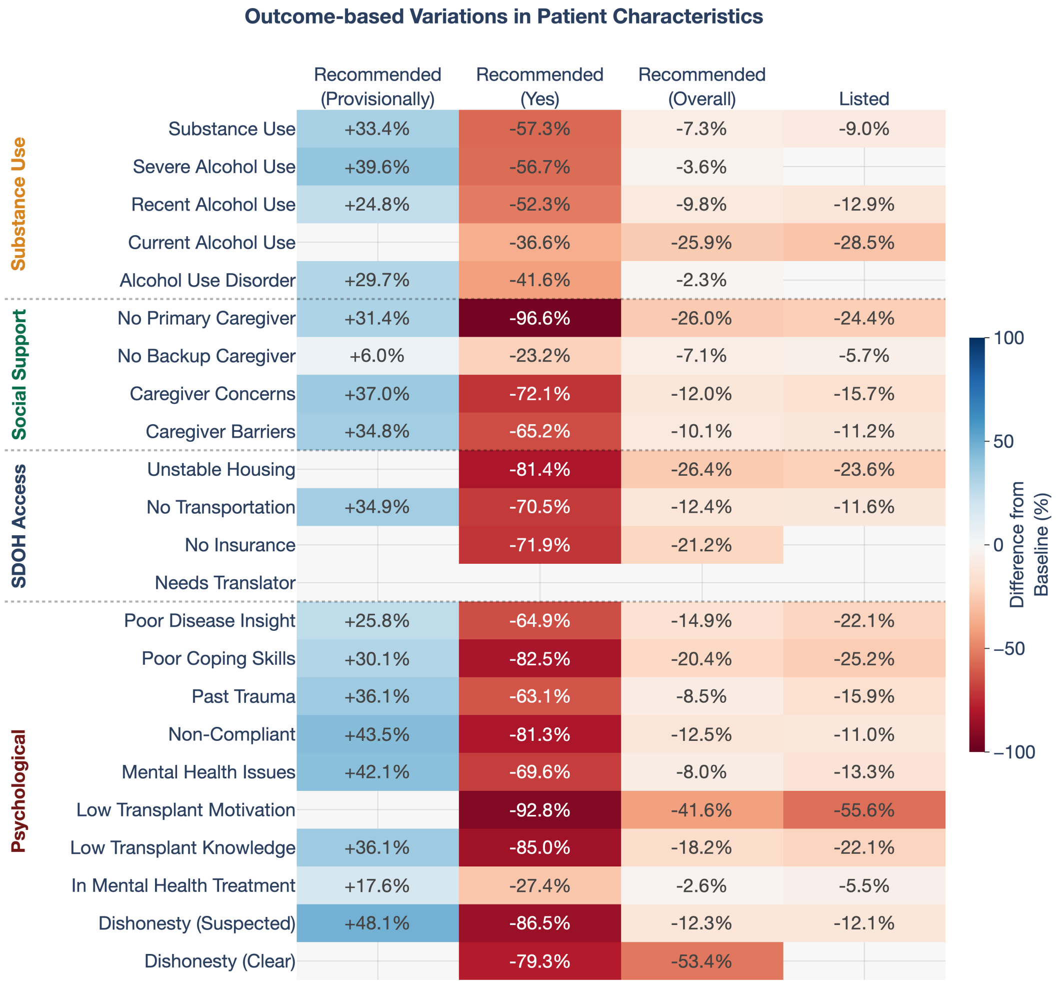

Figure S11: **Psychosocial risk and SDOH and their relationship to expanded psychosocial recommendation designations and listing.** Heat map showing only statistically significant differences in psychosocial risk and SDOH factor prevalence between patients who did versus did not achieve each outcome (two-proportion z-tests, $p < 0.05$, FDR-corrected); blue indicates higher rates, red indicates lower rates, blank cells indicate non-significant differences.

A.3 Extended Data: AUROC Sensitivity Analysis

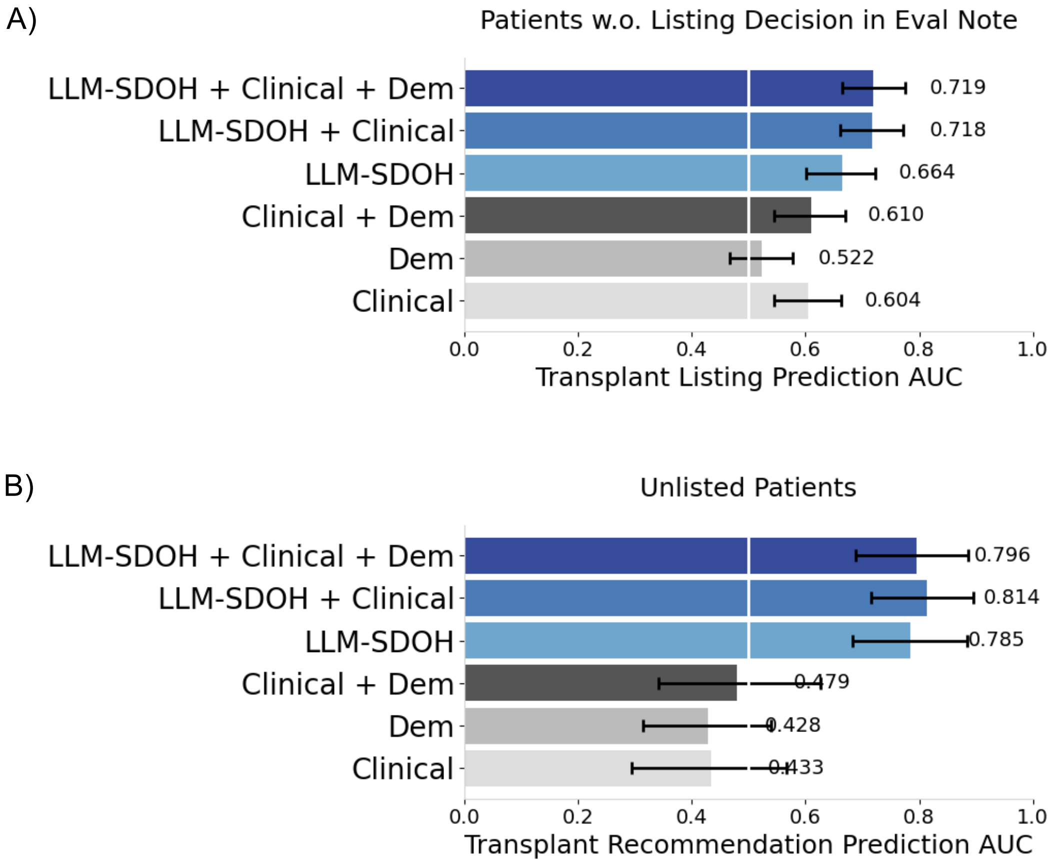

Figure S12: **Sensitivity analysis for data leakage in listing prediction models.** AUROC performance for psychosocial recommendation prediction a) and transplant listing prediction b) restricted to patients whose evaluation notes did not contain listing decision addendums (n=2,947 patients), ensuring temporal separation between feature extraction and outcome determination. Error bars represent 95% confidence intervals. Stable performance compared to full cohort analysis (Figures 4-5) demonstrates that psychosocial risk and SDOH predictive value is not an artifact of data leakage.

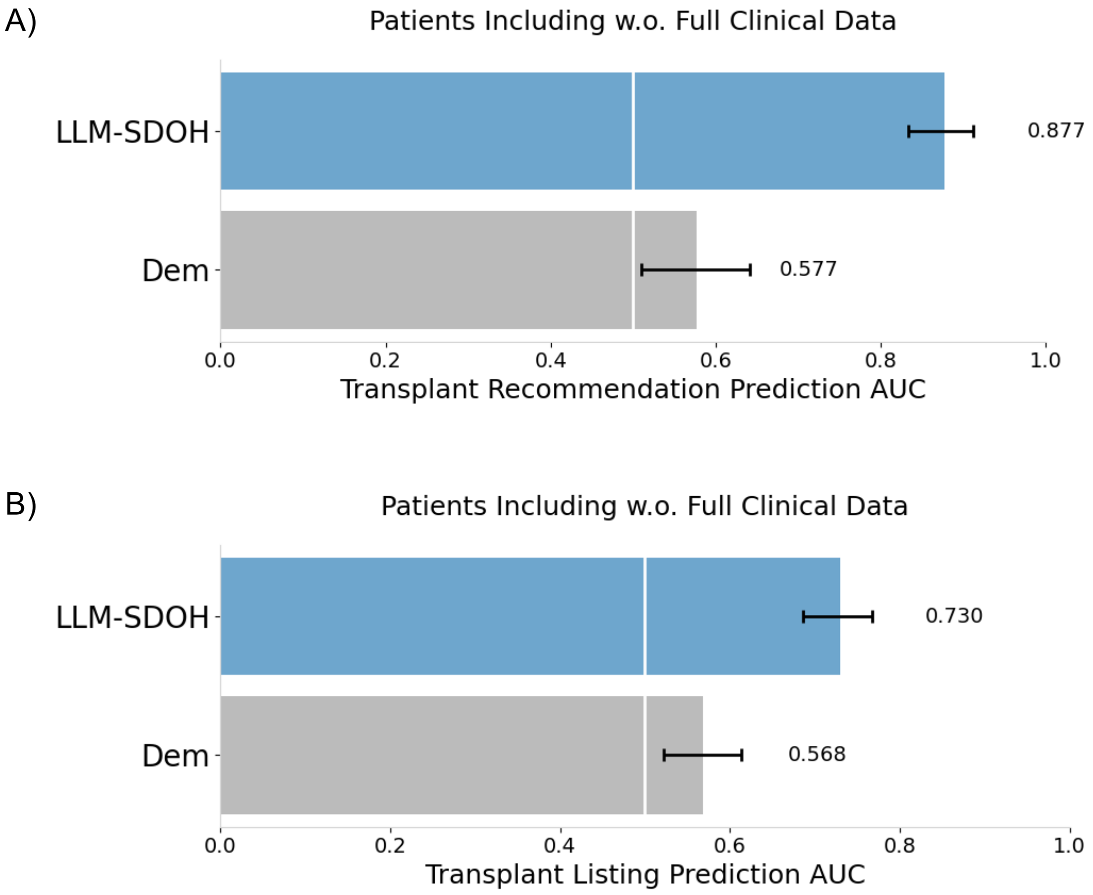

Figure S13: **Sensitivity analysis for missing data bias in predictive model performance.** Comparison of AUROC values for psychosocial recommendation prediction (A) and transplant listing prediction (B) using demographic features alone (Dem) and LLM-derived psychosocial risk and SDOH features alone (LLM-SDOH) in patients with complete clinical data versus expanded cohort including patients with incomplete clinical data (n=548 additional patients). Error bars represent 95% confidence intervals. Stable performance across cohorts indicates minimal bias from excluding patients with missing clinical variables.

A.4 Extended Data: Enhanced Accuracy and Interpretability of LLM-Derived SDOH Features

A.4.1 Text Feature Processing

BOW features were derived from raw note text that was cleaned and preprocessed with the NLTK Python package [?]. Removal of standard stop words and lemmatization with the WordNetLemmatizer was applied prior to count vectorization with the scikit-learn CountVectorizer with an ngram range of 1-2. We filter for terms that appeared in more than five notes, but fewer than 80% of the notes, with a maximum vocabulary size of 10,000 terms. We standardize data with StandardScaler and the top 100 features based on a chi-squared test with the outcome were chosen with the scikit-learn SelectKBest and chi2 implementations. For the cTAKES concepts based model, concepts that appear in >0.1% of notes were selected, counted, and the prevalence was also standardized with StandardScaler.

A.4.2 Detailed Results

Model performance metrics for the XGBoost models based on BOW, cTAKES, or LLM-derived features across the two binary outcomes (recommendation, listing) are summarized in Table S3 with ROC curves shown in Figure S14.

Table S3: Performance of Text-Based XGBoost Models Across Transplant Evaluation Decisions

Outcome	Model	AUROC	Sensitivity	Specificity
Rec. (Overall)	BOW	0.91 (0.89–0.93)	0.92 (0.90–0.94)	0.69 (0.57–0.82)
	LLM	0.87 (0.84–0.89)	0.80 (0.77–0.83)	0.76 (0.64–0.88)
	cTAKES	0.52 (0.49–0.56)	0.50 (0.46–0.54)	0.54 (0.40–0.68)
Listed	BOW	0.71 (0.68–0.74)	0.69 (0.65–0.73)	0.65 (0.57–0.73)
	LLM	0.66 (0.63–0.70)	0.72 (0.69–0.76)	0.50 (0.42–0.58)
	cTAKES	0.54 (0.50–0.58)	0.59 (0.55–0.63)	0.44 (0.35–0.52)

Note: Values shown as mean (95% confidence interval); 0.5 threshold used for sensitivity/specificity calculations. BOW = Bag of Words; cTAKES = clinical Text Analysis and Knowledge Extraction System.

A.4.3 Feature Interpretability Comparison with BOW and cTAKES Features

Here we elaborate on the specific advantages and limitations of the LLM-derived features derived from our approach relevant to their interpretability and utility, with a focus on BOW versus LLM-derived features.

While BOW and LLM-derived features showed similar predictive performance, LLM-derived features offer superior interpretability and utility in evaluating LT decision processes. This is evident from the SHAP values for BOW, cTAKES, and LLM-derived models (Figure S15). LLM-derived features have several advantages over BOW features. They maintain temporal, relational, and negation context, which BOW features often lose. For example, an LLM can accurately answer "Does the patient have any mental health issues that are actively affecting their daily functioning?", while a BOW approach might only capture the presence of the term "mental health issues" without context. LLM-derived features are directly interpretable and designed to capture factors relevant to LT decision-making. For example, the question "What is the severity of the patient's past alcohol use based on the documentation in the note?" provides clear, categorized information (None, Mild, Moderate, Severe, Unknown) that is directly relevant to LT evaluation. In contrast, BOW features may include ambiguous terms like "poor", "lack", "could", or "intact" without clear context or relevance. The LLM approach allows for specification of timing and valence, which is typically unclear in BOW features. For instance, the question "Has the patient used alcohol in the past 6 months?" provides a clear timeframe, while a BOW feature might only indicate the presence of alcohol-related terms without temporal context. Another advantage of LLM-derived features is that they lead to an automatically standardized feature set across all notes, whereas BOW features vary based on note content. This standardization allows for more consistent analysis across patients, with each note being evaluated on the same

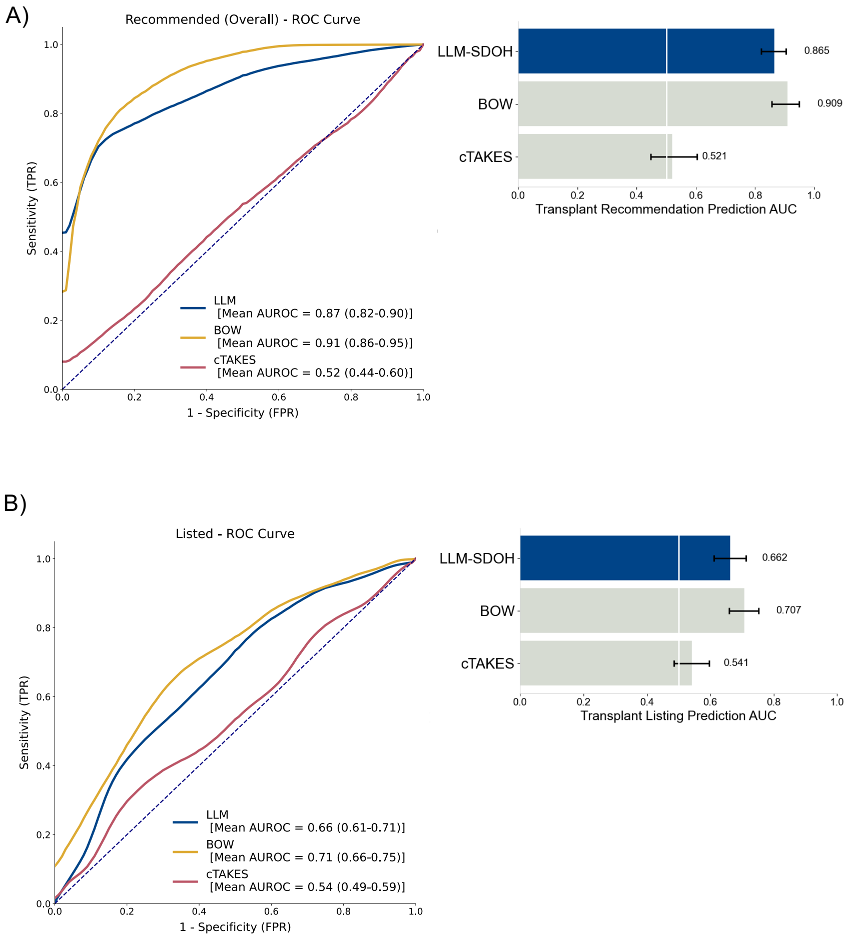

Figure S14: **Predictive Capacity of Text-based Features in LT Process.** a) AUROC curves for XGBoost model performance using LLM-derived, BOW, and cTAKES features to predict psychosocial recommendation (left). Comparison of average AUROC (w. 95% CI) across the feature sets. Feature sets including LLM-derived features highlighted in blue (right). b) Same as (a) but for models predicting transplant listing

set of expert-informed questions. LLM-derived features also capture information at a relevant granularity for LT decision-making. For example, the question "What is the patient's housing situation?" provides categories (Stable Housing, Difficulty Paying for Housing, Without Housing (Undomiciled), Unknown) that are directly relevant to assessing a patient's stability and support system. BOW features, in contrast, might include overly specific details that are less directly relevant to the overall assessment.

However, LLM-derived features have limitations, including possible hallucinations. The way we employ the LLM to essentially "survey" a note introduces concerns similar to survey designs, such as the limitation of only obtaining information we think to ask about and the impact of question wording on extracted information. There's also the possibility that information to answer a particular question may not be mentioned in the note, although this is addressed by including an "Unknown" category in the LLM's response options for each question. The BOW approach, while less targeted, may capture unexpected relevant information. Despite these trade-offs, the interpretability and relevance of LLM-derived features make them particularly useful for analyzing decision points and outcomes in the LT process. In addition, it is worth noting that BOW features may inadvertently include terms directly related to outcomes (e.g., "recommendation", "listing"), potentially leading to label leakage. LLM-derived features can be designed avoid this issue by focusing on patient characteristics rather than process outcomes. They can also be designed to accurately extract such labels that are absent in the structured data-as we do with the psychosocial recommendation-so they can be used (with some caution) as targets for prediction tasks. Overall, the use of LLM-derived psychosocial risk and SDOH features leads to models with similar predictive power as text-based models. However, it's important to note that some of this predictive power in BOW models may come from terms that directly mention recommendation and risk, which are difficult to fully remove from the notes.

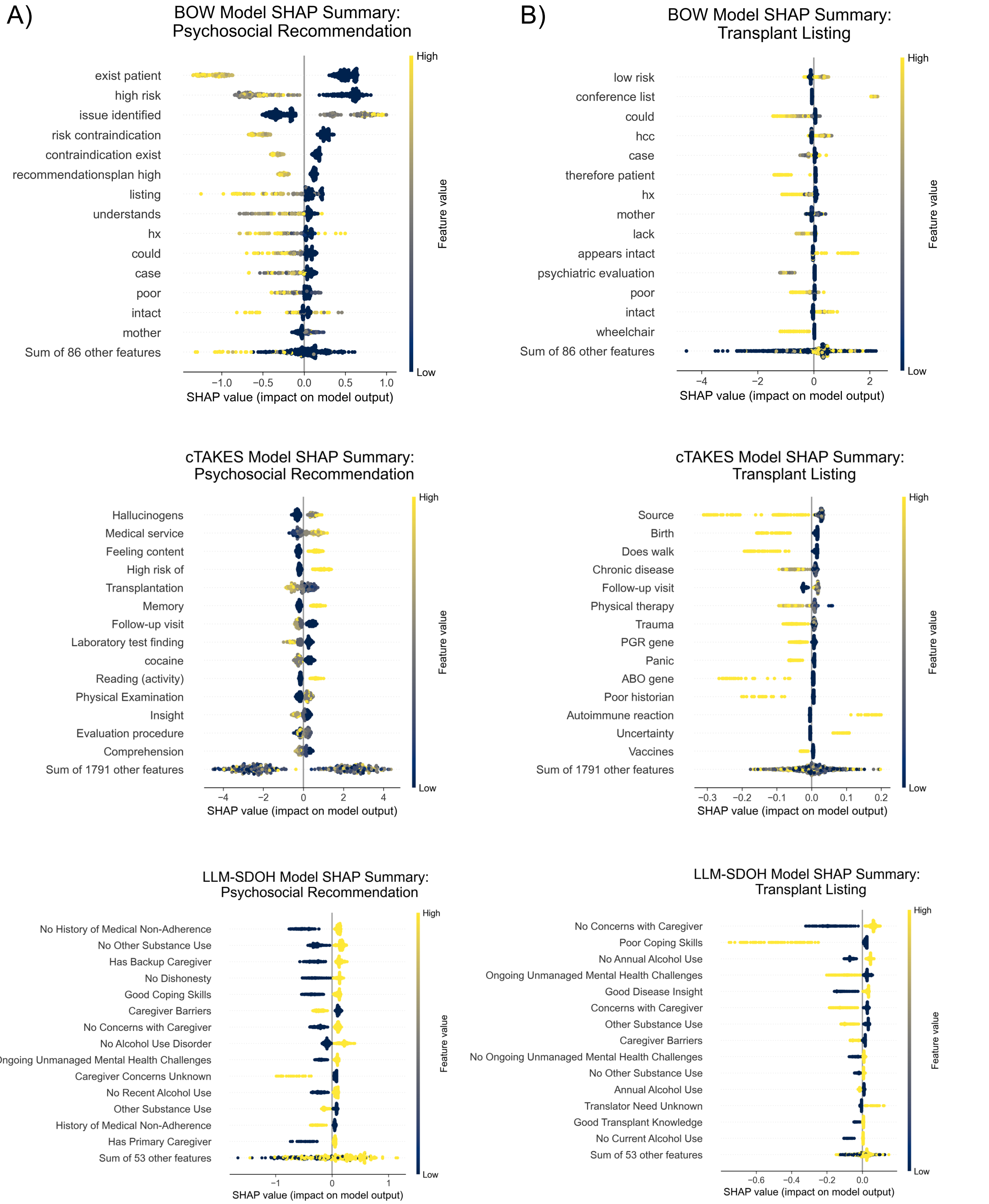

Figure S15: **SHAP Value Summaries for Models Solely Based on Text Features.** a) SHAP values for XGBoost models predicting psychosocial recommendation based on Bag-of-Words (BOW) (top), clinical Text Analysis and Knowledge Extraction System (c-TAKES) (middle), and LLM-derived features (bottom). b) The same as (a) but for models predicting transplant listing. Features ranked by model importance; blue indicates lower feature values, yellow indicates higher feature values.

A.5 Supplementary Tables: SDOH and Psychosocial Factor Summaries

Table S4: **SDOH and Psychosocial Factor Classification and Definitions for Liver Transplant Evaluation.** Complete taxonomy of factors organized by domain (Social Support, Access, Psychological, Substance Use) with factor types and hypothesized modifiability assessments.

SDOH / Psychosocial Factor	Factor Type[a]	Modifiability[b]	Literature Evidence
**SUBSTANCE USE**			
**Current alcohol use** (currentalcohol)	Medical/Clinical	High	OPTN policies and contraindications [14-17]
**Recent alcohol use** (recentalcohol)	Medical/Clinical	High	Transplant protocols and abstinence standards [14-17]
**Severe alcohol history** (alcoholseverity)	Medical/Clinical	Low	Transplant disparities and outcomes [18-19, 30-38]
**Alcohol use disorder** (alcoholusedisorder)	Behavioral/Psychosocial	Moderate	Addiction treatment and management [18-19]
**Substance use (non-alcohol)** (substanceuse)	Medical/Clinical	High	General transplant risk factors [18-19]
**Annual alcohol use** (annualalcohol)	Medical/Clinical	High	Monitoring and treatment protocols [18-19]
**SOCIAL SUPPORT**			
**No primary caregiver** (primarycaregiver)	Socioeconomic/Structural	Moderate	Psychosocial assessment and outcomes [22-23]
**Caregiver concerns** (caregiverconcerns)	Socioeconomic/Structural	Moderate	Psychosocial evaluation factors [22-23]
**No backup caregiver** (backupcaregiver)	Socioeconomic/Structural	Moderate	Support system requirements [22-23]
**Caregiver barriers** (caregiverbarriers)	Socioeconomic/Structural	Moderate	Social determinant interactions [1-2, 21]
**ACCESS**			
**Unstable housing** (housing)	Socioeconomic/Structural	Low	Core social determinants of health [1-2, 21]
**Transportation issues** (transportissue)	Socioeconomic/Structural	High	Healthcare access barriers [1-2, 21]
**Needs translator** (translator)	Socioeconomic/Structural	High	Language access and healthcare quality [26]
**No insurance** (insurance)	Socioeconomic/Structural	Low	Healthcare access barriers [1-2, 21]
**PSYCHOLOGICAL**			
**Poor disease insight** (diseaseinsight)	Behavioral/Psychosocial	High	Health literacy and transplant outcomes [11, 27]
**Low transplant knowledge** (transplantunderstanding)	Behavioral/Psychosocial	High	Patient education and informed consent [11, 27]
**Low transplant motivation** (transplantmotivation)	Behavioral/Psychosocial	Moderate	Psychological readiness assessment [22-23]
**Poor coping skills** (copingstrategies)	Behavioral/Psychosocial	High	Stress management and resilience [22-23]
**Medical non-adherence** (medicalnonadherence)	Medical/Clinical	Moderate	Medication adherence outcomes [24-25]
**In mental health treatment** (mentalhealthtreatment)	Socioeconomic/Structural	High	Mental healthcare access [22-23]
**Mental health issues** (mentalhealth)	Behavioral/Psychosocial	Moderate	Mental health management [22-23]
**Past trauma** (pasttrauma)	Behavioral/Psychosocial	Moderate	Trauma-informed care [22-23]
**Dishonesty (suspected)** (dishonesty)	Behavioral/Psychosocial	Moderate	Patient-provider relationship [22-23]

[a]**Factor Type:** Medical/Clinical = factors directly related to medical care or health behaviors; Behavioral/Psychosocial = individual psychological or behavioral factors; Socioeconomic/Structural = factors determined by social, economic, or structural circumstances. [b]**Modifiability:** High = readily addressable through targeted interventions; Moderate = requires sustained effort or system changes; Low = requires major structural changes.

Classification framework for 23 factors extracted from liver transplant psychosocial evaluation notes using GPT-4-Turbo with 85.9% accuracy, developed through expert consultation with clinical social workers and transplant hepatologists. **Literature Evidence References:** [1-2] Social determinants of health frameworks; [11,27] Health literacy and transplant outcomes; [14-17] OPTN policies and transplant protocols; [18-19] Liver disease disparities and substance use; [21] Social determinants in liver transplantation; [22-23] Psychosocial assessment and SIPAT validation; [24-25] Medication adherence in transplant recipients; [26] Medical interpreter services; [30-38] Transplant outcome disparities.

Table S5: **Clinical Impact and Intervention Planning for SDOH and Psychosocial Factors.** Standardized regression coefficients, prevalence rates, and potential interventions for key psychosocial and SDOH factors affecting liver transplant listing decisions.

SDOH / Psychosocial Factor	Clinical Impact[a]	Key Study Findings	Potential Feasibility[b]	Possible Intervention
		**SUBSTANCE USE**		
**Current alcohol use**	−0.14	2.5% prevalence; listed patients 28.5% less likely to have current use (p=0.02)	High	Addiction treatment, monitoring protocols
**Substance use (non-alcohol)**	−0.05	22.7% prevalence; Non-Hispanic White patients show a 22.1% higher prevalence (p<0.01)	Medium	Substance-specific addiction treatment
**Annual alcohol use**	−0.04	32.7% prevalence; statistically significant predictor (p=0.04)	Medium	Patient education, lifestyle counseling
		**SOCIAL SUPPORT**		
**Primary caregiver presence**	**+0.10**	3.8% lack caregiver; female patients 38.2% more likely to lack caregiver (p=0.03)	High	Caregiver support programs, respite care services
**Caregiver concerns**	−0.08	14.1% prevalence; significant predictor across all models (p=0.01)	Medium	Caregiver training, support groups
		**PSYCHOLOGICAL**		
**Disease insight**	**+0.08**	2.8% have poor insight; good insight positive predictor exceeding MELD score impact (p<0.01)	High	Structured patient education programs
**Past trauma**	−0.07	8.9% prevalence; female patients 36.6% higher (p<0.01)	Low	Trauma-informed care approaches, specialized counseling
**Mental health issues**	−0.06	17.6% prevalence; increase from 2012-2023, female patients 31.6% higher (p<0.01)	Low	Mental health treatment optimization, ongoing support
**Transplant knowledge†**	+0.06	5.6% have poor knowledge; Non-Hispanic White patients show a 27.1% lower prevalence (p=0.07) of knowledge gaps	High	Culturally-tailored educational materials and classes
**Coping skills†**	+0.04	6.6% have poor coping; listed patients 25.2% less likely to have poor coping (p=0.07)	Medium	Stress management counseling, coping skills training

[a]**Clinical Impact:** Standardized regression coefficients from multivariable logistic regression controlling for demographics, MELD score, HCC status, and BMI. Positive values increase listing probability; negative values decrease listing probability. Only p<0.05 shown; †p<0.10. [b]**Potential Feasibility:** High=addressable with existing resources; Medium=sustained coordination required; Low=complex systemic changes needed.

Social determinants extracted using GPT-4-Turbo with 85.9% accuracy (95% CI: 84.6-87.2%) validated against expert annotations. Eight statistically significant predictors identified (p<0.05); two marginally significant factors included for completeness (†p<0.10). Only factors with statistical significance in multivariable models are included to focus on evidence-based targets.
